# Flooding Responses on Grapevine: A Physiological, Transcriptional, and Metabolic Perspective

**DOI:** 10.3389/fpls.2019.00339

**Published:** 2019-03-26

**Authors:** Benedetto Ruperti, Alessandro Botton, Francesca Populin, Giulia Eccher, Matteo Brilli, Silvia Quaggiotti, Sara Trevisan, Nadia Cainelli, Paola Guarracino, Elisabetta Schievano, Franco Meggio

**Affiliations:** ^1^Department of Agronomy, Food, Natural Resources, Animals and Environment, University of Padova, Legnaro, Italy; ^2^Interdepartmental Research Centre for Viticulture and Enology, University of Padova, Conegliano, Italy; ^3^CRIBI Biotechnology Centre, University of Padova, Padova, Italy; ^4^Department of Biosciences, University of Milan, Milan, Italy; ^5^Department of Chemical Sciences, University of Padova, Padova, Italy

**Keywords:** waterlogging, hypoxia, root, transcriptome, gene expression, *Vitis*

## Abstract

Studies on model plants have shown that temporary soil flooding exposes roots to a significant hypoxic stress resulting in metabolic re-programming, accumulation of toxic metabolites and hormonal imbalance. To date, physiological and transcriptional responses to flooding in grapevine are poorly characterized. To fill this gap, we aimed to gain insights into the transcriptional and metabolic changes induced by flooding on grapevine roots (K5BB rootstocks), on which cv Sauvignon blanc (*Vitis vinifera* L.) plants were grafted. A preliminary experiment under hydroponic conditions enabled the identification of transiently and steadily regulated hypoxia-responsive marker genes and drafting a model for response to oxygen deprivation in grapevine roots. Afterward, over two consecutive vegetative seasons, flooding was imposed to potted vines during the late dormancy period, to mimick the most frequent waterlogging events occurring in the field. Untargeted transcriptomic and metabolic profiling approaches were applied to investigate early responses of grapevine roots during exposure to hypoxia and subsequent recovery after stress removal. The initial hypoxic response was marked by a significant increase of the hypoxia-inducible metabolites ethanol, GABA, succinic acid and alanine which remained high also 1 week after recovery from flooding with the exception of ethanol that leveled off. Transcriptomic data supported the metabolic changes by indicating a substantial rearrangement of primary metabolic pathways through enhancement of the glycolytic and fermentative enzymes and of a subset of enzymes involved in the TCA cycle. GO and KEGG pathway analyses of differentially expressed genes showed a general down-regulation of brassinosteroid, auxin and gibberellin biosynthesis in waterlogged plants, suggesting a general inhibition of root growth and lateral expansion. During recovery, transcriptional activation of gibberellin biosynthetic genes and down-regulation of the metabolic ones may support a role for gibberellins in signaling grapevine rootstocks waterlogging metabolic and hormonal changes to the above ground plant. The significant internode elongation measured upon budbreak during recovery in plants that had experienced flooding supported this hypothesis. Overall integration of these data enabled us to draft a first comprehensive view of the molecular and metabolic pathways involved in grapevine’s root responses highlighting a deep metabolic and transcriptomic reprogramming during and after exposure to waterlogging.

## Introduction

During the past decade unprecedented high-impact climate extremes including droughts, heat waves and floods have occurred in all parts of the world (IPCC, 2012, 2013). The frequency of floodings, causing globally more climate-related disasters than any other extreme climate event, increased of about 65% over the last 25 years (FAO et al., 2018). Heavy rainfall events have become on average more intense and more frequent in Europe (Westra et al., 2014). The extent of flooding damage is hardly predictable due to the complex nature of its occurrence, which may significantly vary depending on the amount, intensity, duration and spatial distribution of precipitations, thus making many ecosystems worldwide vulnerable (Bailey-Serres et al., 2012; Shabala, 2012). Furthermore, at least one-tenth (about 12 million ha) of irrigated cropland in the developing world, has lost its productivity due to flooding events (Mancuso and Shabala, 2010; Olesen et al., 2011; Bailey-Serres et al., 2012; Shabala, 2012).

Flooding drastically reduces O_2_ availability for plants’ root respiration and survival (Shabala, 2012), adversely affecting crop production and wild species distribution in natural ecosystems (Bailey-Serres and Voesenek, 2008, 2010). Furthermore, hypoxic conditions exacerbate the competition for oxygen between root and soil microorganisms, thus affecting also the nitrate availability as a consequence of a lower extent of microbiological nitrification (Mancuso and Shabala, 2010).

Deficiency or lack of oxygen in the soil (hypoxia or anoxia, respectively) as well as the overall reducing soil conditions that are generated by anaerobic microorganisms, lead to the accumulation of toxic metabolites (including H_2_S, N_2_, Mn^+2^, Fe^+2^) and ROS (see the list of abbreviations and notations) (Ponnamperuma, 1972), and affect the synthesis of stress hormones (i.e., abscisic acid and ethylene) in roots (Bailey-Serres and Chang, 2005; Miller et al., 2008; Van Breusegem et al., 2008; Lee et al., 2011; Bailey-Serres et al., 2012; Sauter, 2013; Carvalho et al., 2015; Loreti et al., 2016).

Plant adaptation responses to flooding can be classified into two main strategies: the LOQS and the LOES, which hinge on a number of different biochemical and developmental adjustments deeply discussed by Colmer and Voesenek (2009) and Voesenek and Bailey-Serres (2015). A major feature of the LOQS is the reduction of shoot growth, to conserve substrate availability until the water recedes, whereas plants displaying LOES exhibit fast growth of shoot to reach the water surface thus re-enabling gas exchange (Colmer and Voesenek, 2009; Voesenek and Bailey-Serres, 2015). While the LOQS has only been described in lowland tolerant rice varieties, the promotion of shoot elongation by submergence (LOES) is known to occur in wetland and amphibious species over a wide taxonomic range, and in general, in those plants that have adapted to environments prone to temporary shallow floods (e.g., *Rumex palustris*, *Ranunculus sceleratus*, *Nymphoides peltata*, *Potamogeton pectinatus*, and *P. distinctus*) (Ishizawa et al., 1999; Summers et al., 2000; Sato et al., 2002; Voesenek et al., 2004; Mommer et al., 2005).

Grapevine is one of the most widely cultivated plant species worldwide, and in Europe it represents a crop of major economic interest (Anderson and Nelgen, 2011) and is integral to the cultural heritage and landscape (Terral et al., 2010).

The most famous wine-growing regions are located in a narrow geographical area where the best expression of *terroir*, i.e., the optimal combination of environmental and human factors (van Leeuwen et al., 2004), has been tuned through millenary experience. The high specificity of these climatic niches exposes viticulture to the effect of climate change (Kenny and Harrison, 1992; Schultz, 2000; Duchêne and Schneider, 2005; Jones et al., 2005; Santos et al., 2011). The present climate scenario poses new unexpected and urgent challenges to traditional viticulture, that is already threatened and will increasingly face more intense and frequent extreme weather events (Frei et al., 2000; Prosdocimi et al., 2016; Leolini et al., 2018).

Only few studies have been conducted to characterize the effects of flooding events on grapevine’s cultivation. Initial efforts aimed at describing morphological and physiological aspects of grapevine adaptation to waterlogging stress pointed out an overall reduction in stomatal conductance, photosynthetic rate and plant height, as well as premature senescence and disturbances to yield components (Striegler et al., 1993; Stevens and Prior, 1994; Stevens and Harvey, 1995; Kawai et al., 1996; Stevens et al., 1999; Mancuso and Boselli, 2002; de Herralde et al., 2005; Mancuso and Marras, 2006; Mugnai et al., 2011).

In the present study, we aimed to gain insights into the transcriptional and metabolic changes induced by flooding in grapevine roots. A preliminary experiment under controlled hydroponic conditions was aimed at developing a model for the root grapevine response to O_2_ deprivation. Results enabled the identification of transiently and steadily regulated candidate hypoxia-responsive marker genes. Afterward, over two consecutive vegetative seasons, flooding was imposed to vines during the late dormancy period, the most susceptible to high-frequency precipitations events (Crawford, 2003). Transcriptomic and metabolic profiling untargeted approaches were applied on roots to investigate their early molecular and metabolic responses to both the hypoxia and the subsequent recovery after stress removal. Internode elongation was taken as a proxy for low oxygen adaptive responses to hypoxic stress in grapevine and overall data integration enabled drafting a first comprehensive view of the molecular pathways involved in a deep metabolic and transcriptomic reprogramming of grapevine response to waterlogging.

## Materials and Methods

### Plant Material and Treatments

Two experimental set-ups were adopted to study hypoxic responses of grapevine plants using, *Vitis vinifera* L., cultivar Sauvignon blanc (clone 108) grafted on Kober 5 BB (K5BB) (*V. berlandieri* x *V. riparia*) rootstock as experimental material. The first experimental set-up was performed in hydroponics under strictly controlled conditions in a growth chamber while the second one was performed in pots, reproducing a situation similar to that found in the field, in semi-controlled conditions under a tunnel located at the Experimental Farm of the University of Padova “L. Toniolo” in Legnaro, north-east of Italy. K5BB was chosen as a rootstock in all the experiments, since it is one of the most widely distributed rootstocks across european viticulture and it is known to display a moderate tolerance to flooding stress.

#### Hydroponic Experiment

Preliminary experiments were conducted to identify potential grapevine hypoxia-responsive genes. To this end, six vines were grown in 5 L pots in hydroponic solution (modified Hoagland medium) and arranged under two experimental conditions: three control plants were maintained in a constantly oxygenated solution and three plants were maintained in a solution without oxygenation and isolated from ambient air by adding a vaseline layer in order to prevent oxygen diffusion into the solution and enable progressive oxygen depletion by root respiration.

O_2_ concentration (mg/L) was monitored in the solution using a portable multiparametric probe (HQ40d HACH, Loveland, CO, United States) and roots were sampled after 12, 24, and 96 h after stress imposition, during an experimental period of 6 days.

#### Pot Experiments

Pot experiments were carried out over two consecutive years (2016 and 2017) to achieve extensive transcriptome and metabolic profilings to characterize grape response to flooding. Three years-old plants were grown in 10 L pots filled with a sand–pumice-peat mixture (2:2:6 in volume). Sixty plants were selected each year based upon homogeneous developmental characteristics (i.e., length of the cane and number of buds), pruned before bud burst by retaining three or four latent buds per plant, and arranged under the following two experimental conditions at the phenological stage BBCH05 (“Wool stage” according to Lorenz et al., 1995): (i) control (C), plants that were maintained at 80% of soil field capacity, and (ii) flooded (F), plants that were flooded by inserting the pots in wider containers filled with tap water, so that the water level was maintained 5 cm above soil surface. During an experimental period of 21 days, measurements of dissolved O_2_ concentration (mg/L) and flooding water temperature were conducted using a portable AquaPlus (Aquaread water monitoring instruments) (Supplementary Figure S1). Sampling of plant material was carried out in both years at 2 (T2), 8 (T3), 16 (T4), and 21 (T5) days after stress application. In 2017, an additional sample was collected at 1 day (T1) after flooding application to pinpoint early responses to flooding. After the last sampling, the water was withdrawn from the pots and after 7 days (28 days after stress application, T6) an additional sampling of material was performed (Supplementary Figure S1). For each time point and sampling, the roots of five plants for both control (C) and stress (F) conditions were gently washed with water to remove soil and the young roots were immediately dissected, frozen in liquid nitrogen and stored at −80°C for following analyses. The roots of each plant were kept separate representing a single biological replicate, for a total of five independent biological replicates for each experimental condition and time-point.

Samples and biological replicates for RNA-Seq analyses of both years were selected on the basis of the time-course and on the relative level of expression of the hypoxia marker genes *VvACO1*, *VvSUS4*, and *VvADH1*, identified as described below.

### RNA Extraction, cDNA Synthesis, and RT-qPCR

Total RNA was extracted from 80 mg of young roots using the “Spectrum^TM^ Plant total RNA Kit” (Sigma, St. Louis, MO, United States) according to manufacturer’s instructions. DNase treatment was performed using the On-Colum Dnase I Digest set (DNASE70, Sigma, St. Louis, MO, United States) during the RNA purification. Total RNA was quantified with the NanoDrop 2000c (Thermo Scientific, Waltham, MA, United States) and its integrity was checked by running 200 ng in a 1% agarose gel stained with SYBER^®^ Safe (Life Technologies, Carlsbad, CA, United States). cDNA was synthesized with the SuperScript^®^ VILO^TM^ cDNA Synthesis Kit (Life Technologies, Carlsbad, CA, United States) from 500 ng of DNA-free total RNA in a final volume of 20 μl, according to the instruction provided by the manufacturer. RT-qPCR was performed using StepOne Real-Time PCR System (Applied Biosystems, Monza, Italy) as described by Nonis et al. (2008) and Eccher et al. (2015), using SYBR Green reagent (Applied Biosystems, Monza, Italy). The analysis was performed on three independent biological replicates for each treatment by using specific primers listed in Supplementary Table S1. Gene expression levels were calculated using the automated Excel spreadsheet Q-Gene designed by Simon (2003), using the modification of the ΔCt method suggested by Pfaffl (2001). Gene expression values were normalized with *V. vinifera* genes encoding for *VvUBC28* published by Castellarin et al. (2007) and reported as arbitrary unit of Mean Normalized Expression.

*Vitis vinifera* genes encoding two 1-ACC oxidases (*VvACO2* and *VvACO1*), an *VvADH1* and a *VvSuS4* were identified (Jaillon et al., 2007; Vitulo et al., 2014) as candidate hypoxic marker genes, on the base of the general involvement of their homologs from model plants in plants’ responses to hypoxia and anoxia (Licausi et al., 2011; Bailey-Serres et al., 2012). Their low-oxygen-dependent expression in grapevine was confirmed by comparing RNAs obtained from control and O_2_-deprived roots from the hydroponic experiment.

### RNA-Seq Analyses

#### Genome and Annotation

The revised version of the Grape genome available at http://genomes.cribi.unipd.it/DATA/ and the corresponding gff file were used as a reference in all the experiments. The genome is based on the 12X Genoscope Pinot Noir genome (Jaillon et al., 2007), while the annotation has been later on revised by extensive use of RNA-seq data (Vitulo et al., 2014). GO functional annotation was also downloaded from the same location and used for all enrichment analyses as described below.

#### RNA-Seq 2016

Total RNA was sent to the center Genomix4life (University of Salerno, Salerno, Italy) for quality check, libraries preparation, and sequencing. Libraries were sequenced by using an Illumina NextSeq 500 platform (Illumina, San Diego, CA, United States) obtaining ∼33 millions of stranded single-end reads of 50 base pairs per sample.

RNA-seq reads were checked for quality using FastQC (Brabaham Bioinformatics, Cambridge, United Kingdom), resulting of average very high quality. Adapter content was negligible and the reads were not trimmed before mapping. Sequence duplication levels were moderate, and since for single-end reads the removal of duplicates can give problems (Klepikova et al., 2017), the entire set of reads was mapped. Spliced alignments were performed with TopHat (Trapnell et al., 2009) and only uniquely mapping reads (identified by the flag NH:i:1 in the bam alignment file) were used for transcript abundance quantification. Quantification was performed by using the Bedtools coverage program (Quinlan and Hall, 2010) and the gff file.

#### RNA-Seq 2017

Samples from 2017 were sequenced with a different protocol called mRNA QuantSeq FWD by Lexogen (Lexogen GmbH, Vienna, Austria) and therefore were also analyzed differently, as in this case there is an accumulation of reads at the end of the transcripts. Libraries were sequenced by using an Illumina NextSeq 500 platform (Illumina, San Diego, CA, United States) allowing to obtain ∼20 millions of stranded single-end reads of 75 base pairs per sample. More in detail, QuantSeq FWD reads were trimmed by following the instructions given by the manufacturer^1^ and mapped with TopHat. Transcript abundance was quantified as explained above.

The raw sequence reads data files of both years were uploaded to the SRA database with BioProject ID PRJNA521303.

#### Differential Expression Analysis

Genes undergoing differential expression in treated Vs untreated samples were identified by using the DESeq2 package (Love et al., 2014). Lowly expressed genes were filtered in a homogeneous way for all libraries, by first normalizing the counts into RPKM, and then all transcripts with an average expression below 1 RPKM were removed from the counts matrix to be used for differential expression analysis. In this way, the number of tests performed by DESeq2 to detect differential expression prior to *p*-value adjustment was reduced. Significantly changing transcripts were defined at an alpha of 1E-04 and having at least a doubling/halving of their expression level in the two contrasted conditions. The FDR was fixed at 5E-05. The FDR threshold was kept very stringent, as the *p*-value correction is made on a per contrast basis, but the analyses were performed on a high number of different samples (Supplementary Tables S2, S3).

#### Enrichment Analysis

Functional enrichment of GO categories was obtained by exploiting the available GO annotation of the Grapevine genome. For this objective, the GOStats package (Falcon and Gentleman, 2007) was used in R (R Core Team, 2018). Enrichments of Biological Processes were calculated separately for the up and down regulated genes identified as differentially expressed, at a FDR level of 0.05.

### Metabolites

#### Sample Preparation

Five independent biological replicates of each treatment (from control or flooded roots, season 2017) and time point (T1, T2, and T6) were used for ^1^H-NMR analysis. Metabolites were extracted from 30 mg of freeze-dried powder of young roots using a 1:1 (v/v) mixture of D_2_O (in 400 mM phosphate buffer, pH = 6.00) and Methanol-*d4*, >= 99.8 atom % D (Sigma-Aldrich) in a final volume of 1 ml, and mixed briefly by vortexing for 15 s. Samples were sonicated for 30 min at 20°C and then centrifuged at 9000 rpm for 5 min.

A solution of 15 mM Calcium formiate (Ca(HCOO)_2_ (Sigma-Aldrich TraceCERT, product no. 03826) was prepared in D_2_O to be used as internal standard for the quantification of metabolites. For each sample, 593 μl of the supernatant were transferred to a NMR tube and 7 μl of the standard solution were added, leading to a 0.18 mM concentration of internal standard in the tubes.

#### NMR Parameters

A Bruker DMX 600 spectrometer, operating at a proton frequency of 599.90 MHz, was used for the ^1^H-NMR experiments. The magnetic field was locked to the MeOH-*d*_4_ resonance. The spectra were recorded using NOESY pulse sequence with a water presaturation (noesy1dpr in Bruker notation), 256 transients of 32 K points, spectral width of 14 ppm, relaxation time of 2 s, acquisition time 25 min, at 298 K.

To provide quantitative data, a reference sample has been acquired with an additional relaxation time of 60 s to ensure complete spin relaxation of the nuclei in all metabolites. In this way the metabolites concentration of this sample was determined. A relaxation time (T_1_) correction factor was calculated by comparing the signal absolute value of each metabolite in the reference spectra acquired with 2 and 60 s of relaxation delay, respectively. The concentration of the metabolites in all the other extracts was obtained by using this correction factor.

The ACDlab v.12.5 software was used to process FIDs and spectra. The FIDs were multiplied by an exponential window function with 0.5 Hz line broadening and zero-filled to 64K points. The spectra resulting from Fourier transformation were manually phased and baseline-corrected. The MeOH-*d*_4_ signal was used to calibrate the frequency.

#### Data Pre-processing and Statistical Analysis

Each ^1^H spectrum was bucketed and integrated using intelligent bucketing of intervals of 0.035 ppm. The spectra were normalized to the total sum of integral covering the interval 9–0.5 ppm (excluding the solvent signal regions). Pareto scaling and mean centering were applied before multivariate data analysis. The obtained data set resulted to be composed of 240 variables.

The data matrix was analyzed using the pattern recognition methods in the SIMCA software (version 14.0, Umetrics, 2015) including unsupervised PCA and OPLS-DA. The quality of the discriminant models was evaluated by the goodness of fit score (R^2^Y) and the goodness of prediction score (Q^2^Y). The S-plot (Wiklund et al., 2008) was used to identify the characteristic resonances (significant based on both the *t*-test for the means and of the Mann-Whitney test for the medians, *p* < 0.001) responsible for classes distinction.

### Biometrical Measurements

Once bud-break occurred, internode elongation rate was measured on ten primary shoots per treatment from the lower side of one node to the lower side of the node above it using a graduated ruler on a weekly basis throughout all the vegetative season.

Internode extension in grapevine shoot has been described being sigmoidal over time in four stages (Lebon et al., 2004; Louarn et al., 2007). These are: Stage I, during which elongation is exponential; Stage II, which is short and during which the extension rate increases rapidly; Stage III, during which the extension rate is essentially constant and internode length increases linearly; and Stage IV, during which the extension rate decreases as the internode approaches its final length. Internodes have been arranged in 6 classes starting from the basal to the apical ones: 1–2 (class I), 3–5 (class II), 6–10 (class III), 11–15 (class IV), 16–20 (class V), and 21–25 (class VI). For each class, final internode length, internode elongation duration and maximum internode elongation rate were estimated by non-linear least square regression of the change in internode length on a DOY basis, using a three-parameter Gompertz sigmoidal model as follow:

(1) $$\mathrm{Internode length}=a\cdot e^{-e^{-\left(\frac{x-x_{0}}{b}\right)}}$$

where *a* is the asymptote corresponding to final internode length, *x*_0_ is the *x*-value at the inflection point corresponding to the midway point in the duration of internode expansion, and *b* is the maximal slope at the inflection point, corresponding to maximal internode elongation rate. The values and confidence intervals of these parameters were estimated with Sigmaplot software. No R^2^ was lower than 0.98 and no standard error of estimate was higher than 0.15 (data reported in Supplementary Table S4). For each internode class, the first derivative from the sigmoidal growth functions, representing the AGR were computed in order to assess the duration of growth and their rates in term of cm per day. At the ‘5 separated leaves’ stage (stage 15 according to the extended BBCH scale; Lorenz et al., 1995), the plants were thinned to two branches and were trained vertically.

## Results

### Identification and Validation of Low Oxygen Stress Markers in Grapevine Roots

**FIGURE 1:**
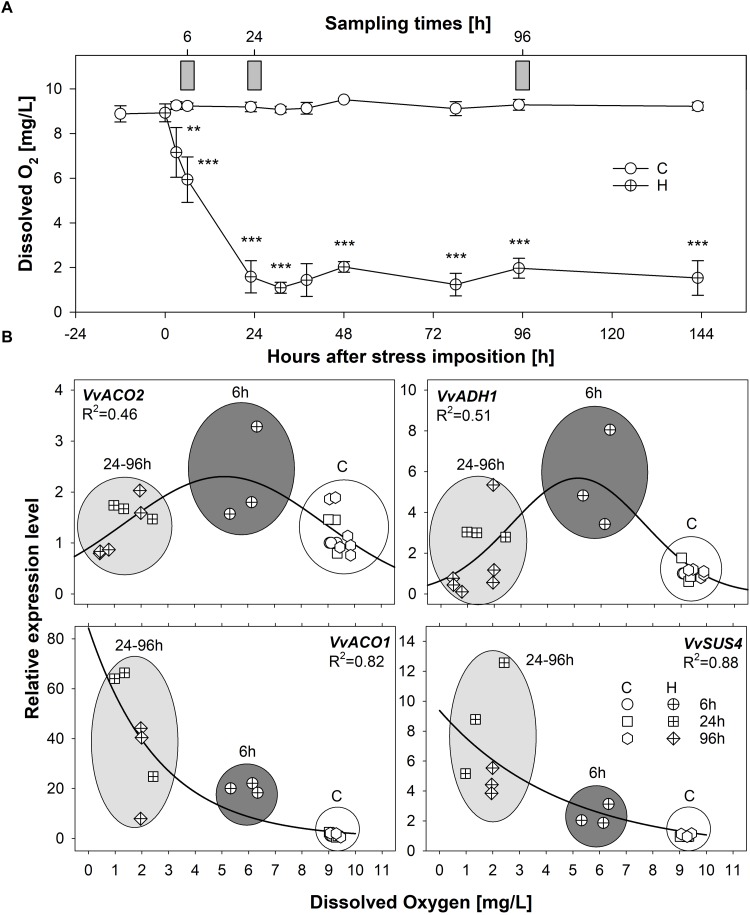
**(A)** Levels of dissolved oxygen (mg/L) in hydroponic solution, measured in the vicinity of roots during a time-course experiment of 6 days in 5 L beakers constantly oxygenated (control plants; empty circles) or not oxygenated and isolated from oxygen (plants exposed to hypoxia; crossed circles); values represent mean ± SE and asterisks denote mean values that are statistically different at ^∗∗^*p* < 0.01, ^∗∗∗^*p* < 0.005; gray bars indicate sampling points for gene expression analyses of hypoxia-responsive marker genes shown in panel **B**. **(B)** Scatter plot of the expression of the candidate hypoxia-responsive markers *VvACO1* and *VvACO2*, *VvADH1* and *VvSuS4* plotted against levels of dissolved oxygen in hydroponic solution under well oxygeneted (empty symbols) and progressive hypoxic conditions (crossed symbols). Gray-shaded ellipses/circles indicate the expression levels measured at 6 h (dark gray) and 24–96 h /light gray); indicate the expression levels measured at successive time-points; white circles group the expression levels measured in roots of control plants at all time-points. Solid lines indicate three-parameter Gaussian and hyperbola decay best fit curves for (*VvACO2*, *VvADH1*) and (*VvACO1, VvSuS4)*, respectively. Supplementary Figure S2 enables the statistical identification of significant differences among the samples under the different Control and Flooded treatments.

Due to the absence of published studies on hypoxic responses in grapevine roots, putative candidate low oxygen responsive marker genes for grapevine were initially identified on the base of similarity searches, leading to the selection of genes encoding two ACC oxidases (*VvACO1* and *VvACO2*), one *VvSuS4* and one *VvADH1*, respectively known to mark hypoxic responses in a wide range of species of both model and crop plants (Licausi et al., 2011; Bailey-Serres et al., 2012). A preliminary experiment was carried out to correlate the oxygen levels in the solution and the expression of hypoxia responsive marker genes in roots of grapevine plants grown in hydroponics. Significant differences in terms of dissolved O_2_ concentration could be detected between control and hypoxic conditions already after 6 h from stress imposition (Figure 1A). The concentration of dissolved O_2_ dropped to less than 2 mg/L after 24 h from the start of application of stress and remained very low throughout the experiment. The transcriptional profiles observed for the selected genes (*VvACO1* and *VvACO2*, *VvADH1* and *VvSuS4*) showed an induction in response to oxygen levels lower than 8 mg/L thus confirming their hypoxia-dependent regulation (Figure 1B). More in detail, *VvACO2* and *VvADH1* were transiently induced after 6 h followed by a subsequent down-regulation after 24 and 96 h of O_2_-deprivation, while *VvACO1* and *VvSuS4* showed a progressive trend of increase of transcription along the duration of stress condition, with a maximum accumulation of transcripts after 96 h (Supplementary Figure S2).

### Transcriptional Responses of Flooded Grapevines

*VvACO1*, *VvSuS4*, and *VvADH1* genes were used as markers to select reproducible and representative biological replicates for further RNA-Seq (both in 2016 and 2017) and metabolic analyses (in 2017). Their expression was higher in flooded roots than in roots of control plants in all the four independent biological replicates considered (Supplementary Figure S3). Differently from roots of plants grown in hydroponics, all genes displayed a transient induction at T1 that leveled off at later time-points in both years. Measurements of the oxygen levels dissolved in the free solution of pots confirmed that flooded plants experienced a drop in oxygen availability that roughly halved at day 1 (T1) from stress imposition in comparison to control plants, reaching 4.6 mg/L and 5.7 mg/L in 2016 and 2017, respectively. Already at day 2 (T2), flooded plants experienced a drop in oxygen availability that reached values of 2.9 mg/L and 4.4 mg/L in 2016 and 2017, respectively. A further drop in oxygen availability was observed at day 16 (T4) with values around 2 mg/L that settled until the end of the flooding (day 21) for both years analyzed (Supplementary Figure S1). Based on the kinetics of both oxygen levels and marker gene expression, three out of four independent replicates were selected for further untargeted molecular analyses among the samples collected in both 2016 and 2017 at T2 (hypoxia) and T6 (1 week of recovery to normal conditions). An additional time point at day 1 (T1, early hypoxia) was also included in 2017 analyses to gain more insight into the early molecular responses to low oxygen.

The RNA-Seq transcriptomic profiles observed for flooded and control roots evidenced very similar and nearly overlapping transcriptional signatures for the different biological replicates and for both the years analyzed (Supplementary Figure S4).

Our results overall pointed out that the same groups of genes were consistently up- or down-regulated in both years (Supplementary Figure S5) even though with slightly different dynamics especially for the up-regulated ones. A clearly identifiable group of genes appeared to be collectively up-regulated in flooded plants at T2 in 2016 (Supplementary Figure S6A), while appeared to undergo a two step regulation in 2017 with some of them showing a maximum induction either at T1 or T2 (Supplementary Figure S6B). In addition, the expression of such genes appeared to return to levels comparable to those of control plants at T6 (Supplementary Figure S6).

Overall, RNA-Seq analyses led to the *bona fide* identification of groups of genes that are reliably and consistently regulated as a consequence of exposure to flooding and, therefore, to a low oxygen stress in grapevine roots (K5BB).

### GO Enrichment and KEGG Pathway Analyses of Hypoxia-Regulated DEGs in Grapevine Roots

Analyses of enriched GO terms (biological process) for genes that were up- or down-regulated in response to flooding showed large similarities with results previously achieved in other species in response to low oxygen availability (Blokhina et al., 2003; Mustroph et al., 2010). Several GO terms resulted to be significantly enriched in both seasons analyzed and are listed in Table 1 with their statistics for both T1 and T2 of year 2017. GO terms enriched in up-regulated genes included categories related to oxygen depletion and oxidative stress such as “response to hypoxia” and “response to oxygen levels”, “oxygen transport”, “regulation of hydrogen peroxide”, and “ROS metabolic process”, as well as the GO categories related to carbon and nitrogen metabolism such as “carbon utilization”, “gluconeogenesis”, and “nitrate assimilation” (Table 1). A different time-course of transcriptional regulation could be observed among the GO functional groups, with some of them being significantly enriched at both time points, while others at either T1 or T2.

**Table 1:** GO terms, for the “biological process” dictionary, that were enriched (*p* < 0.001; FDR < 0.05) at either T1 or T2 (or both) in 2017, in up- (red block) and down- (blue block) regulated genes.

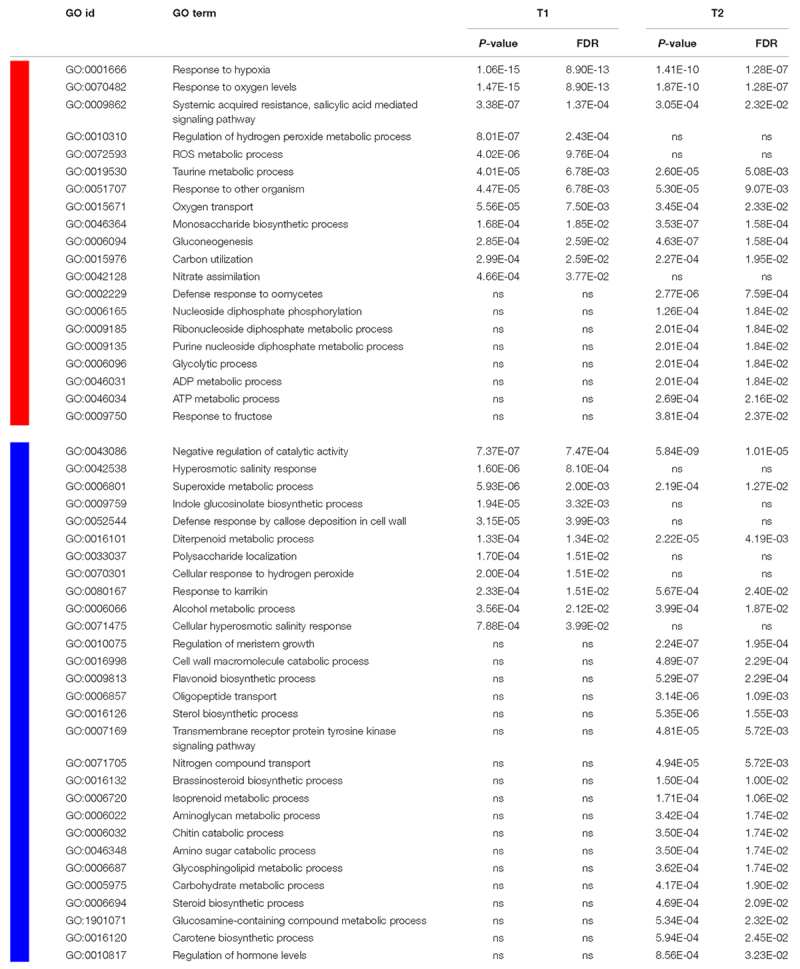

Only the terms enriched also in 2016 are shown.

Among GO categories enriched in down-regulated genes in response to hypoxia, those related to hormone biosynthesis and, in particular, to “sterol”, “steroid”, and “brassinosteroid biosynthetic process” and those related to “isoprenoid” and “diterpenoid metabolic process” as well as “carotene biosynthetic process” and “regulation of hormone levels” appeared to be significant in both years. GO functional categories involved in catabolic and growth processes, such as “regulation of meristem growth” and “cell wall, chitin and amino sugar catabolic processes”, resulted to be enriched as well.

### Primary Metabolism: Glycolysis and TCA Cycle

Based on the assignment of the identified DEGs on KEGGs Maps several transcriptional changes could be assigned to well-defined metabolic pathways, among which primary metabolism appeared remarkably impacted. Considering the higher temporal resolution of the 2017 RNA-Seq analysis, due to the inclusion of an additional time-point at day 1 (T1) from the start of flooding, and the overall comparability between the transcriptomic profiles in the two seasons, for simplicity further in depth data elaboration has been focused on the 2017 season only.

**FIGURE 2:**
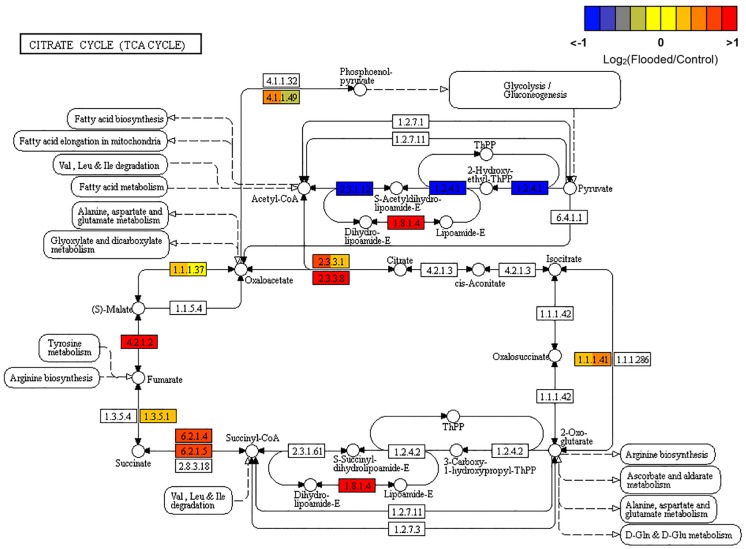
KEGG map of the Citrate Cycle (TCA cycle; map00020) pathway, rendered by Pathview (Luo et al., 2017). Each box is divided in three sub-boxes and represents a specific enzymatic step with the relative expression of the corresponding genes in Flooded versus Control roots, calculated as log2-ratio at T1, T2, and T6 (from left to right).

Hypoxia is known to determine a complete rearrangement of primary metabolism to ensure cell survival in oxygen shortage, by hijacking sugar metabolism from the tricarboxylic acid (TCA) cycle, which is inhibited and reversed, toward an increased glycolytic flux. In hypoxic grapevine roots the induction of the genes responsible for the conversion of citrate to oxaloacetate (citrate synthase [EC:2.3.3.1], of malate to fumarate (fumarate hydratase, class I [EC:4.2.1.2]) and, to a minor extent, of fumarate to succinic acid (SDH, [EC:1.3.5.1]) and of the ATP citrate-lyase [EC:2.3.3.8]), are coherent with the above described reversion of the reactions of the Citrate Cycle, which results in the accumulation of succinate in response to hypoxia (Bailey-Serres et al., 2012). Succinic acid formation appeared to be further sustained through the activation of the gene encoding the alpha subunit of succinyl-CoA synthetase [EC:6.2.1.5] (Figure 2).

**FIGURE 3:**
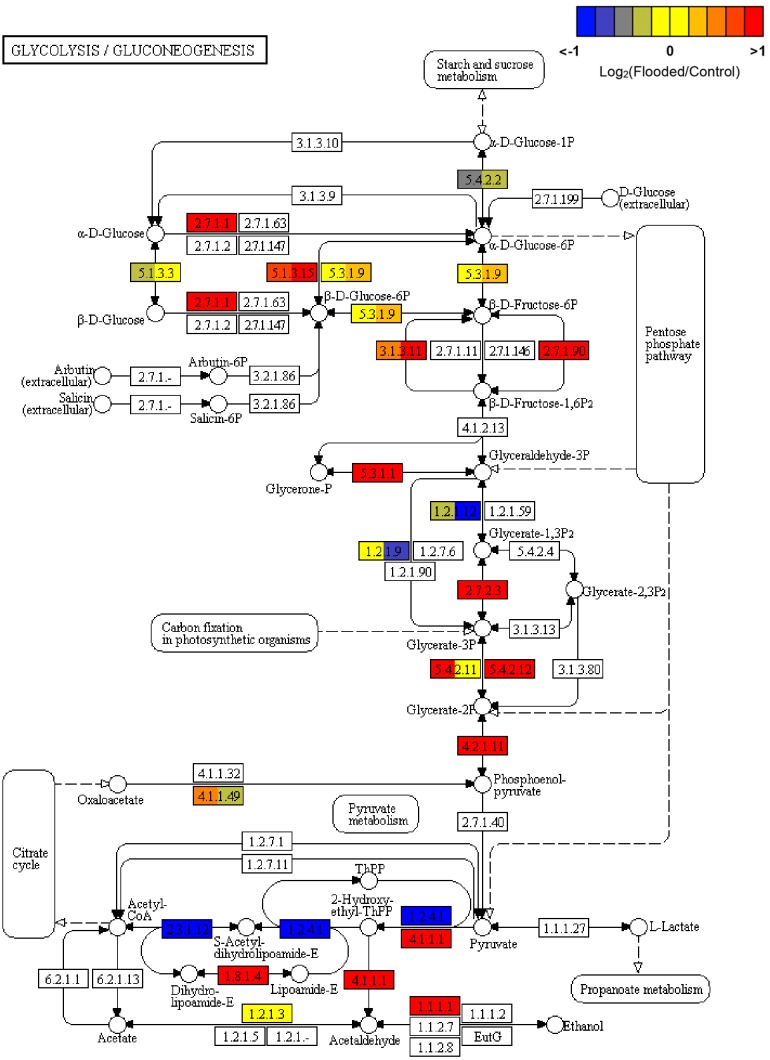
KEGG map of Glycolysis and Gluconeogenesis (map00010) pathway, rendered by Pathview (Luo et al., 2017). Each box is divided in three sub-boxes and represents a specific enzymatic step with the relative expression of the corresponding genes in Flooded versus Control roots, calculated as log2-ratio at T1, T2, and T6 (from left to right).

Consistently with the inhibition of the TCA cycle, leading to acetyl-CoA shortage in hypoxic cells, fatty acids biosynthesis and elongation appeared overall inhibited (data not shown). Regarding glycolysis, the transcriptomic data from flooded grapevine roots indicated that phosphoglucomutase [EC:5.4.2.2] was down regulated, probably to reduce flux through gluconeogenesis, while all genes encoding enzymes involved in the glycolytic and fermentative pathways were induced. These genes included hexokinase [EC:2.7.1.1] as well as glucose-6-phosphate 1-epimerase [EC:5.1.3.15], the latter involved in the conversion of beta-D-glucose 6-phosphate into alpha-D-glucose 6-phosphate, thus enhancing the channeling of glucose-6P into glycolysis. The stable induction of diphosphate-dependent phosphofructokinase [EC:2.7.1.90] in the absence of changes in the transcripts encoding the ATP dependent 6-phosphofructokinase 1 [EC:2.7.1.11], both responsible for the glycolytic flux maintenance through the conversion of D-fructose 6-phosphate to D-fructose 1,6-bisphosphate, is a hallmark of the typical metabolic shift taking place in response to low oxygen, by the stimulation of PPi-dependent rather than ATP-dependent processes as an energy saving mechanism. Interestingly, a gene encoding fructose-1,6-bisphosphatase I [EC:3.1.3.11], leading to regeneration of D-fructose 6-phosphate and phosphate was also induced. Further flooding/hypoxia-induced genes in grapevine roots controlling key steps in glycolysis included phosphoglycerate kinase [EC:2.7.2.3], 2,3-bisphosphoglycerate-dependent and -independent phosphoglycerate mutase [EC:5.4.2.12 and EC:5.4.2.12, respectively] and enolase [EC:4.2.1.11], overall leading to increased later formation of pyruvate, a central hub for the alcoholic fermentation pathway. This latter pathway, appeared consistently up-regulated by the co-ordinated transcriptional activation of PDC [EC:4.1.1.1] and *VvADH1* [EC:1.1.1.1], controlling the formation of acetaldehyde and ethanol, respectively (Figure 3).

**FIGURE 4:**
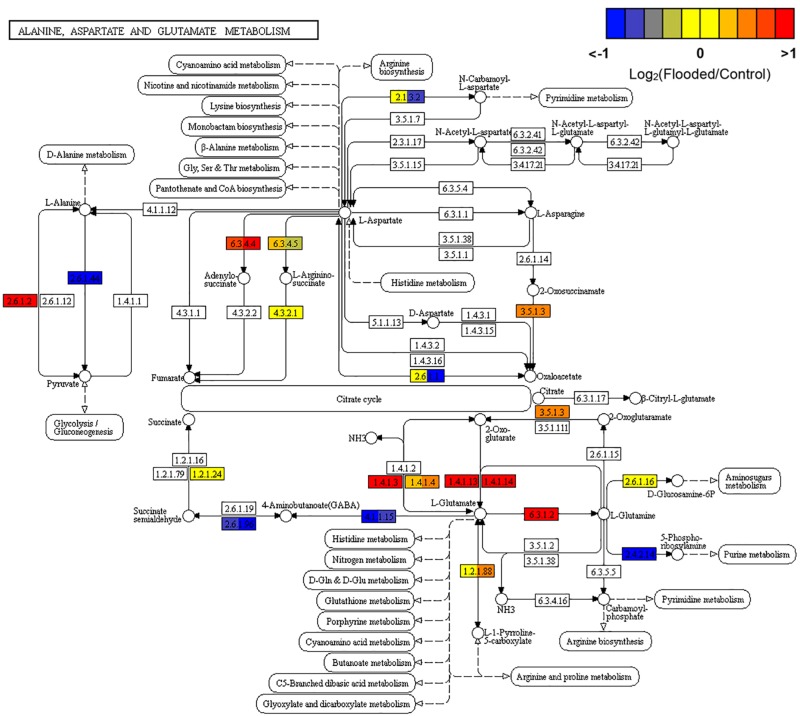
KEGG map of Alanine, Aspartate and Glutamate metabolism (map00250) pathway rendered by Pathview (Luo et al., 2017). Each box is divided in three sub-boxes and represents a specific enzymatic step with the relative expression of the corresponding genes in Flooded versus Control roots, calculated as log2-ratio at T1, T2, and T6 (from left to right).

Overall these data confirm previous reports showing consistently an augmented flux through glycolysis as the preferential pathway to provide metabolic energy while ensuring recycling of reduced NADH, through PPi-dependent and ATP-independent pathways and through the activation of the ethanolic fermentation in which PDC and ADH play a key role. In parallel to the induction of the fermentative pathway, a number of genes involved in amino acid metabolism (Reggiani, 1999) evidenced an overexpression during low oxygen exposure of grapevine roots: alanine aminotransferase (alanine transaminase [EC:2.6.1.2]), responsible for the conversion of pyruvate to alanine, glutamate dehydrogenase [EC:1.4.1.3], converting glutamic acid to 2-oxoglutarate, and glutamate synthase [EC:1.4.1.13 and EC:1.4.1.14], re-converting 2-oxoglutarate into glutamate, the latter further metabolized to glutamine by the induction of glutamine synthetase [EC:6.3.1.2]. The steps involved in the synthesis and metabolism of GABA shunt (glutamate decarboxylase [EC:4.1.1.15] and 4-aminobutyrate-pyruvate transaminase [EC:2.6.1.96], respectively) appeared to be down-regulated (Figure 4).

### Quantification of Key Primary Metabolites Confirmed the Transcriptomic Analyses

**FIGURE 5:**
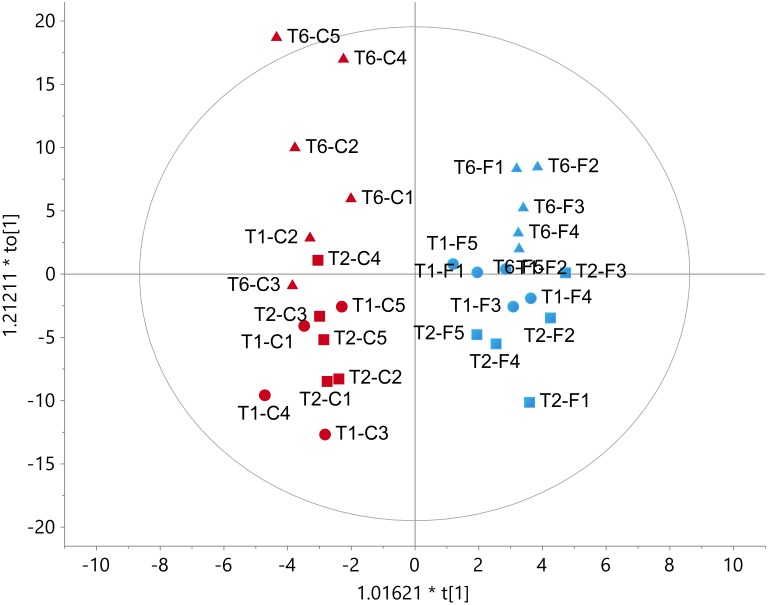
Score scatter plot for the OPLS-DA model on ^1^H-NMR spectra for D_2_O/ MeOH-*d*_4_ roots extracts of flooded (F, blue symbols) and control (C, red symbols) plants in five biological replicates (C1 to C5 and F1 to F5) at the three time points considered (R^2^Y = 0.93, Q^2^Y = 0.27). Solid cicles (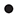): T1; Squares (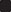): T2; Triangles (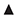): T6.

^1^H-NMR data clearly showed the effects of time and treatment on the metabolic profile of the roots as it can be observed in the score scatter plots of the OLPS-DA model reported in Figure 5. The x axis (t[1]) enabled a clear separation of flooded roots from control ones (Figure 5), underlying a prevailing effect of the hypoxic response (exemplified by the segregation of all flooded samples with respect to the control into the positive and negative sides of the *x* axis of Figure 5, respectively). The effect of time/development (described by the distribution of samples at different time-points along the *y* axis, to[1]rpm) on the overall NMR-detectable metabolic profile appeared similar in both control and flooded roots. Some differences could be shown at T6, when flooded samples resulted to be more similar to their T1 and T2 counterparts while T6 control samples evidenced a wider distribution (Figure 5).

**FIGURE 6:**
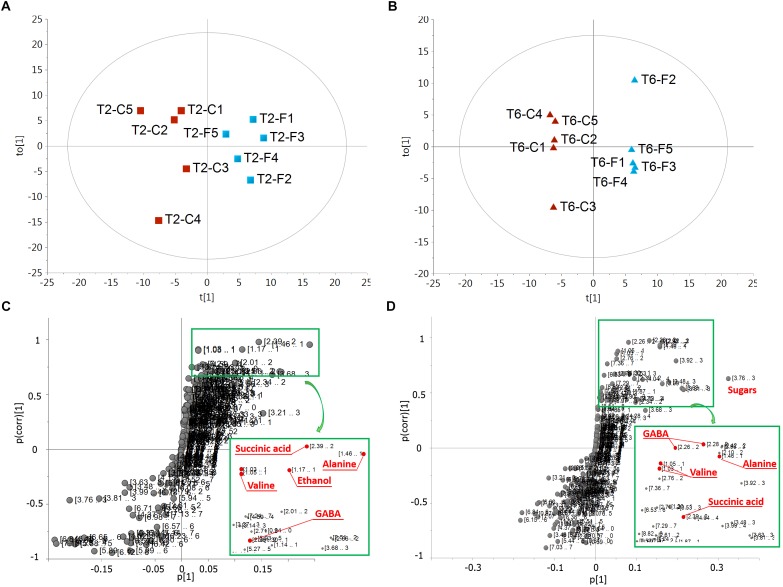
Scores **(A,B)** and corresponding S-plot **(C,D)** of orthogonal partial least-squares-discriminant analysis (OPLS-DA) on ^1^H-NMR spectra for extracts of control **(C)** and flooded **(F)** samples at the T2 (**A**,**C**, R^2^Y = 0.87, Q^2^Y = 0.77) and T6 (**B**,**D**, R^2^Y = 0.99, Q^2^Y = 0.89) time point, respectively. Square insets in the S-plots on the bottom right highlight the metabolites mainly contributing to the separation of flooded samples.

**FIGURE 7:**
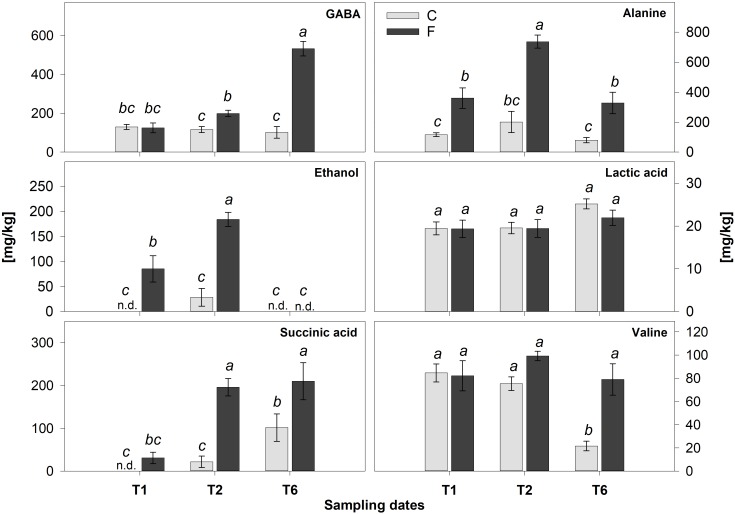
Quantification of the main metabolites identified by ^1^H-NMR in flooded (F) and control (C) samples at T1, T2, and T6. Values are mean ± SE (*n* = 5). Significant differences (Duncan’s test, *p* < 0.05) are marked by different letters, n.d., not detectable.

The prevalent metabolic shift appeared specifically associated with the hypoxic stress, responsible for the separation of flooded samples is highlighted by the OPLS-DA models performed on the T2 (Figure 6A,C) and T6 (Figure 6B,D) replicates, respectively. The insets of the S-plots in Figure 6C,D show the characterizing resonances of flooded samples together with the metabolite assignment of some of them. The profiles of these metabolites in control and flooded roots over the time are reported in Figure 7. The concentration levels of known hypoxia inducible metabolites, such as ethanol, succinic acid, alanine and gamma-aminobutyric acid (GABA) increased in flooded samples (Rocha et al., 2010; Bailey-Serres et al., 2012; António et al., 2016; Figure 6). These differences were particularly evident at T2 (Figure 6A) and leveled off 1 week after the recovery from flooding (T6) for some metabolites, including ethanol which became undetectable (Figure 6B, 7). On the contrary, GABA, succinic acid, alanine and valine levels remained significantly higher at T6 in samples that had previously experienced flooding with respect to control ones (Figure 6B, 7).

### Rearrangement of Hormonal Homeostasis and Action as a Response to Flooding

**FIGURE 8:**
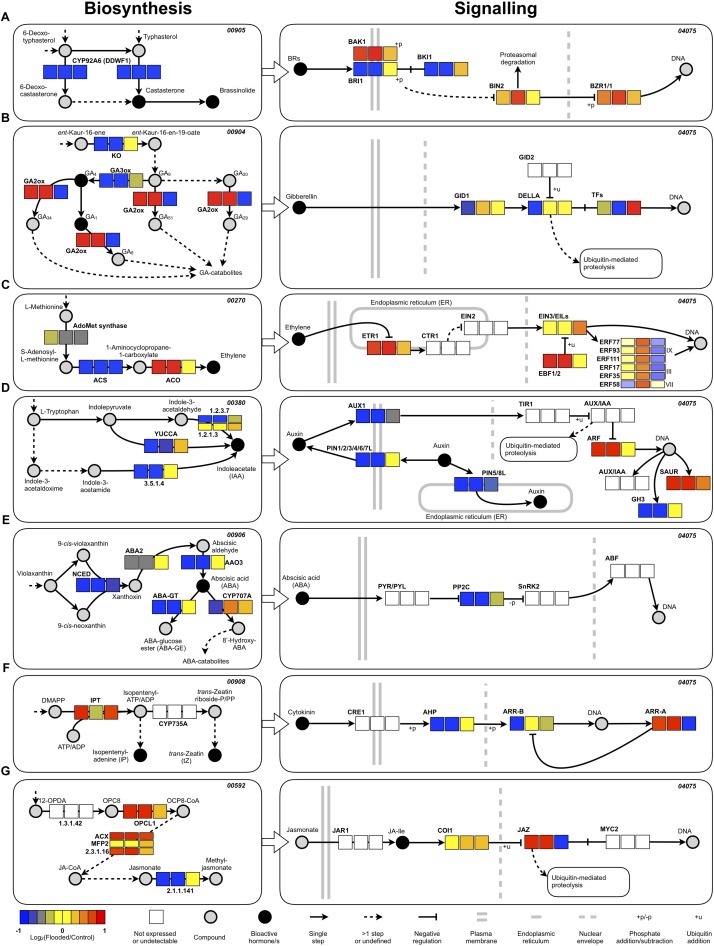
Expression patterns of the most relevant genes related to hormone biosynthesis/homeostasis, perception, and signal transduction plotted within the correspondent KEGG maps (the pathway code is also indicated at the top-right of eachmap). Only the most relevant part of the pathways of brassinosteroids **(A)**, gibberellins **(B)**, ethylene **(C)**, auxin **(D)**, abscisic acid **(E)**, cytokinins **(F)**, and jasmonate **(G)** were extracted (the whole maps are available as Supplementary Figures S7–S14) and represented according to the standard KEGG visualization, with the same colors rendered by Pathview (Luo et al., 2017) and some slight variations (an additional legend is provided): the names of the compounds and the key enzymes/proteins are reported with regular and bold fonts, respectively. Concerning the ERFs, only those with consistent expression patterns in both years are reported along with their nomenclature (Licausi et al., 2011) and classification. The differential expression level was calculated as log2-ratio (Flooded versus Control), scale-centered for each time point, and color-coded as displayed in the legend (blue: <–1; red: >+1). Each box, representing a specific enzymatic or regulatory step, is divided in three sub-boxes representing the different time points: from left to right T1, T2, and T6.

GO analysis of RNA-Seq data pointed out a significant enrichment of the “regulation of hormone levels” category, suggesting a significant rearrangement of hormonal homeostasis of grapevine roots in response to flooding and during recovery thereafter. Specific assignment of DEGs to the respective KEGG hormone biosynthesis and signal transduction maps pinpointed an overall down-regulation of ethylene and ABA biosynthesis during waterlogging (T1 and T2) through a lower accumulation of transcripts encoding the enzymes involved in the respective rate limiting steps: ACC synthase (ACS or ACC synthase) for ethylene (Ju and Chang, 2015; Figure 8C) and NCED and AO3 for ABA (Schwartz et al., 2003; Figure 8E). For the latter hormone, this effect was also paralleled by an increased conjugative and degradative metabolism, witnessed by higher transcript levels of genes controlling ABA glycosylation (ABA-GT) and hydroxylation (CYP707A) steps. A partial re-activation of ABA biosynthesis and metabolism was evident during the recovery phase (T6). As far as the signaling pathways of these two hormones are concerned, an overall negative regulation of ethylene signaling was evidenced by the co-ordinated up-regulation of genes encoding ethylene receptors (ETR) and EBF 1/2 factors (Binder et al., 2007), both exerting an inhibitory action on downstream responses of the canonical ethylene signal transduction pathway (Ju and Chang, 2015). On the other hand, analysis of the grapevine genes encoding ERF ARR factors enabled the identification of three group IX, two group III and one group VII ERF encoding genes that were up-regulated during waterlogging (T1 and T2) and later down-regulated during recovery (T6) (Figure 8C, bottom right inset). An overall down-regulation of genes encoding key biosynthetic limiting steps was evident for brassinosteroids (CYP92A6 encoding genes), gibberellins (ent-kaurene oxidase encoding genes) and auxins (YUCCA and indole-3-acetaldehyde oxidase encoding genes) during waterlogging. The down-regulation of auxin biosynthesis was paralleled by the transcriptional repression of genes encoding both influx (AUX1) and efflux (PINs) carrier proteins (Figure 8D) and GH3 proteins, while ARFs and SAUR encoding genes were up-regulated. Remarkably the down-regulation of GA biosynthesis was accompanied by a parallel up-regulation of degradative metabolic hydroxylation (GA2ox encoding genes) during waterlogging (T1 and T2) while both these effects were reversed during recovery (T6) (Figure 8B) suggesting a reappraisal of biosynthesis and a reduction of metabolism during this latter phase. As far as cytokinins are concerned, an up-regulation of biosynthetic genes (IPT) was present, while general negative regulators of signaling (type A ARR factors) (To et al., 2004) appeared up-regulated and positive regulators (AHP and type B ARR factors) (Mason et al., 2005) appeared up-regulated during flooding, an effect that was reversed during recovery (Figure 8F). Finally, an up-regulation of genes involved in key steps of jasmonic acid synthesis (OPCL1; ACX; MFP2) was evident at all time points, while a clear up-regulation at T1 and T2, followed by down-regulation at T6, was found for the negative regulators of jasmonic acid responses (JAZ) transcriptional repressors (Chini et al., 2016; Figure 8G).

### Differential Effects of Flooding on Grafted Shoot Elongation in Relation to Internode Position Along the Plant Axis

**FIGURE 9:**
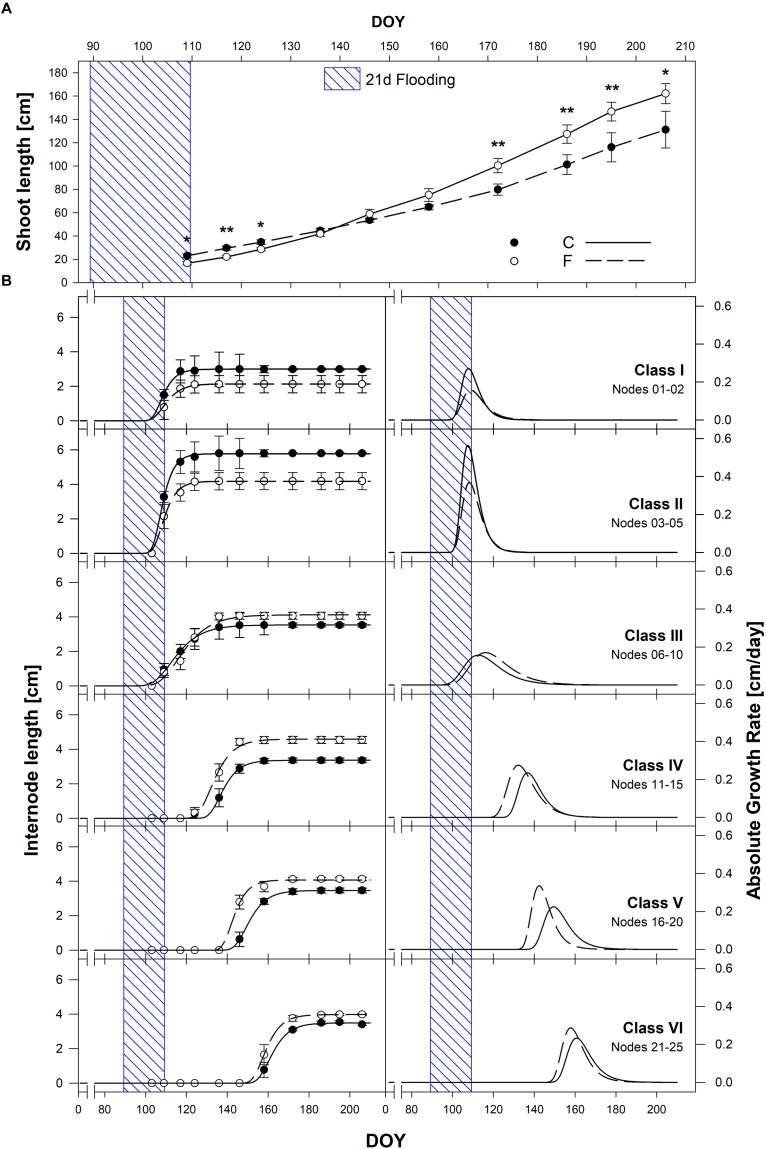
**(A)** Shoot length growth dynamics among (C, black circles) and flooded (F, white circles) treatments throughout the 2017 season. **(B)** Mean internode classes elongation dynamics: 1–2 (class I), 3–5 (class II), 6–10 (class III), 11–15 (class IV), 16–20 (class V), and 21–25 (class VI). Sigmoidal curves represent best fitting curves through non-linear regression for C (solid line) and F (dotted line) treatments enabling AGR dynamics computation for each internode class. Asterisks denote mean values that are statistically different at ^∗^*p* < 0.05, ^∗∗^*p* < 0.01.

Shoot length varied significantly as a result of internode extension kinetics under C and F conditions in season 2017 (Figure 9). The same pattern and significant differences among treatments were present in season 2016 (Supplementary Figure S15). In both seasons, bud-break occurred during the last days of flooding, leading to a significant initial reduction of shoot length in flooded plants. This inhibitory effect lasted about 20 days after stress removal. After that, a marked recovery on shoot elongation of F plants was observed leading initially, to a similar shoot length among treatments (after 2 months from the end of the stress), and to significantly longer shoots in F plants compared to C at the end of the season, with average shoot length of about 170 cm and 130 cm for F and C plants, respectively (Figure 9A). For a deeper understanding of these growth dynamics, the growth of single internodes was measured throughout the season (Figure 9B). To do so, internodes have been arranged in 6 classes of internodes starting from the basal to the apical ones: 1–2 (class I), 3–5 (class II), 6–10 (class III), 11–15 (class IV), 16–20 (class V), and 21–25 (class VI) and their elongation dynamics were computed during both seasons (Figure 9B, season 2017; season 2016, Supplementary Figure S15). Upon recovery, the mean length of F internodes resulted significantly shorter of about 23 and 33%, and maximum growth rates were lower of about 42 and 34%, than C ones for class I and II, respectively. The growth dynamics of class III internodes resulted to be similar both in timing of development, maximum length and elongation rates. On the contrary, median to apical F internode classes IV, V and VI (11–15, 16–20, and 21–25 nodes) resulted longer (19.6, 15, and 18%), with higher (17.4, 49.3, and 23.1%) and earlier (1.1, 4.4, and 4.2 days) elongation rates compared to C ones (Figure 9B).

## Discussion

The past decade has seen an astonishing run of record-breaking storms, forest fires, droughts, heat waves, and floods around the world with just 1.0°C of global warming (IPCC, 2018). More frequent flooding events due to heavier precipitations are expected worldwide in the context of climate change posing new challenges to traditional viticulture.

The most important constraint that plants have to deal with during flooding is O_2_ deficiency. The drastic reduction of O_2_ availability consequent to waterlogging impacts plant metabolism and, in turn, crop growth and productivity. In depth studies have shed light on the low oxygen adaptive responses in different models, both crop and wild plants (Licausi and Perata, 2009; Bailey-Serres et al., 2012; Loreti et al., 2016). The information on grapevine (*Vitis spp*) responses to waterlogging is scattered and basic information on molecular and metabolic responses of grapevine roots to hypoxia is lacking. Such studies are further complicated by the fact that cultivated grapevines are hybrid plants resulting from different rootstock x scion combinations thus making it necessary to take into account complex interactions between different genotypes and the environment. Clarifying how the metabolic events taking place in the below ground organs (rootstock) may co-ordinate or influence the development of above ground organs (scion), during and after waterlogging stress, must necessarily consider different genotypic combinations. Pioneering studies have reported various degrees of tolerance to flooding for some grapevine rootstock genotypes obtained by inter-specific crosses of American varieties: ‘K5BB’ and ‘420A’ (*V. berlandieri* x *V. riparia*), ‘3309C’ (*V. riparia* x *V. rupestris*) and ‘1616C’ (*V. longii x V. riparia*) have been reported as moderate flooding tolerant compared to more sensitive ones such as ‘41B’, ‘110 R’, ‘140Ru’, and ‘1103 P’ (*V. berlandieri* x *V. rupestris*) (Striegler et al., 1993; Kawai et al., 1996; de Herralde et al., 2005; Mugnai et al., 2011). Such differences may be at least in part ascribed to the different adaptive behavior to hypoxia and/or anoxia of the parental genotypes *V. riparia* (originating from riverbanks and thus tolerant to short term flooding) and *V. rupestris* (originating from arid areas and flooding sensitive). *V. riparia*’s higher tolerance has been functionally connected to its better ability to maintain ion homeostasis (sustaining K^+^ uptake) during prolonged hypoxia and to preserve enough O_2_ for the respiratory needs in the root apical meristem (Mancuso and Boselli, 2002; Mancuso and Marras, 2006; Mugnai et al., 2011). As far as the coordinated development of the below and above ground organs is concerned, previous studies reported that root flooding occurring in coincidence with the onset of budbreak did not suppress budbreak but rather inhibited shoot and later root growth (Striegler et al., 1993; Stevens and Prior, 1994; Kawai et al., 1996; Mancuso and Boselli, 2002; Mancuso and Marras, 2006; Mugnai et al., 2011). So far, short term (up to 2 weeks) single or repeated cycles of waterlogging have been shown to cause growth reductions in grapevine, in terms of shoot elongation, leaf growth, altered nutrient composition and leaf gas exchange (Striegler et al., 1993; Stevens and Prior, 1994; Stevens and Harvey, 1995; Stevens et al., 1999; de Herralde et al., 2005).

In the present study, we aimed at obtaining the first molecular and metabolic characterization of the low oxygen responses of the moderate flooding tolerant K5BB rootstock on which *V. vinifera* cv. Sauvignon blanc vines had been grafted. Over two consecutive vegetative seasons, a long term flooding stress was imposed for 21-days to vines at the late dormancy period preceding budbreak. The rate of internode elongation was measured as a proxy for rootstock to scion signaling during the 95 days following the end of the flooding stress. This experimental set-up is peculiar in that the flooding stress was imposed in a period preceding budbreak when grapevine roots had started to be metabolically active but plants did not have shoots and leaves to compensate for oxygen deficiency in roots. This choice was made in order to mimic exactly the timing at which the most frequent flooding events occur in the field (IPCC, 2012, 2013). Genome-scale RNA-Seq transcriptomic analyses have been integrated with ^1^H-NMR metabolic profiling to pinpoint molecular and metabolic changes taking place at the root level in time-course experiments at 1 day (T1) and 2 days (T2) of flooding as well as after 1 week from the recovery from flooding (T6, 28 days) (Supplementary Figure S1).

Indeed, transcriptomic data consistently pointed out a significant primary metabolic reprogramming of K5BB grapevine roots in response to flooding already after 1 (T1) and 2 (T2) days from the onset of stress, showing the up-regulation of a set of genes involved in the enhancement of the glycolytic and ethanolic fermentative pathways, the partial reversal of the TCA cycle and a substantial change in amino acid metabolism (Figure 2–4). These gene expression changes were supported by metabolic data showing significantly increased accumulation of ethanol (but not of lactic acid), succinic acid, alanine and GABA, respectively, in flooded roots in comparison to control ones (Figure 7). These data are in agreement with a metabolic adjustment typical of a low oxygen quiescent strategy (LOQS). LOQS is typical of plants experiencing long periods of exposure to flooding or submergence for which a higher survival rate is ensured by energy saving strategies based on substantial metabolic reprogramming (Bailey-Serres et al., 2012). This consists in a shift toward fermentative metabolism coupled with an increase in the glycolytic flux and with changes in amino acid metabolism. In LOQS, the accumulation of metabolites such as lactate, ethanol, succinate, and even malate witnesses the rewiring of plant cell primary metabolism and the activation of hypoxia-inducible metabolic pathways (van Dongen and Licausi, 2015; Paul et al., 2016). Increased levels of alanine and GABA are specific landmarks of low oxygen conditions (Ricard et al., 1994; Reggiani, 1999) and underlie the substantial change in metabolism of amino acids in response to anoxia, for which a central role is played by glutamate, the common precursor of both alanine and GABA (Branco-Price et al., 2008). Indeed, a recent work by António et al. (2016), implementing an earlier work by Rocha et al. (2010), has provided evidence showing that the increase in GABA, alanine and succinic acid in response to hypoxia is manly a consequence of the activation of the GABA shunt and of the inhibition of the conversion of succinate to fumarate by SDH. Our metabolic and transcriptomic data are in agreement with such findings. Our data, besides confirming such metabolic shifts in response to hypoxia also pointed out that in K5BB grapevine roots the accumulation of GABA and succinic acid, differently from that of ethanol that can diffuse out of the roots, tends to persist also after 1 week of recovery.

The mechanisms involved in the low oxygen sensing and transduction, underpinning the LOQS metabolic shifts, have been largely clarified (Gibbs et al., 2011; Licausi et al., 2011) and sets of specific genes up- or down-regulated in plants in response to flooding and low oxygen have been identified (Loreti et al., 2016).

A prominent role in oxygen sensing and signal transduction in plants has been attributed to the ERF ARR factors belonging to group VII, which behave as master regulators of at least a subset of low oxygen responses (Gibbs et al., 2011; Licausi et al., 2011). The proteins encoded by these genes are subject to a finely tuned post-translational regulation, being degraded in the presence and stabilized in the absence of oxygen, thus leading to their migration into the nucleus and to the activation of the transcription of the LOQS adaptive genes. Our transcriptomic and metabolic data show that K5BB grapevine roots, possibly due to the parental contribution of *V. riparia*, undergo the typical metabolic changes that represent a hallmark of a quiescent metabolic state. These data may support the hypothesis that K5BB or, more in general, grapevine rootstocks with a moderate tolerance to flooding events (e.g., those including *V. riparia* as a parent) (Striegler et al., 1993; Kawai et al., 1996; de Herralde et al., 2005; Mugnai et al., 2011) may base their low oxygen adaptation strategy on a quiescent (LOQS) rather than on an escape behavior (LOES). Among the known grapevine AP2/ERF ARR factors family (Licausi et al., 2010) expressed in K5BB roots we could identify a single gene encoding a *bona fide* group VII ERF protein that appeared to be transcriptionally up-regulated during hypoxia (T2) and later down-regulated during recovery (T6). Whether this gene may be the one ERF responsible for part of the adaptive metabolic responses evidenced in this work on hypoxic K5BB roots will need to be further investigated.

Ethylene plays a primary role in the regulation of hypoxic responses (reviewed by Sasidharan et al., 2018) also through the regulation of AP2/ERF genes. However, the transcriptomic profiling of flooded K5BB roots pointed out substantial transcriptional changes connecting several hormonal biosynthetic and signal transduction pathways and suggesting that an entire rearrangement of the hormonal balance takes place in response to hypoxia as a consequence of reciprocally dependent cross-talks (Figure 8). A general down-regulation of brassinosteroids, auxin and gibberellin biosynthesis appeared evident during waterlogging (T1 and T2). These changes may pinpoint decreased auxin levels in hypoxic K5BB roots, also associated with the down-regulation of both influx (AUX1) and efflux (PINs) auxin carriers, known to be connected with the intracellular auxin content and involved in the determination of its flux (Band et al., 2014). On the other hand, an up-regulation of transcription of ARF genes, positive regulators of auxin responses, was also seen in the absence of regulation of AUX/IAAs. Even though an univocal conclusion on the regulation of auxin responses cannot be drawn, these data may complement those recently reported by Eysholdt-Derzsó and Sauter (2017) showing the hypoxia-induced and ethylene- and RAP2.12-dependent down-regulation of auxin transport through a decreased protein abundance of the auxin efflux carrier PIN2, leading to increased auxin responses in hypoxic *Arabidopsis* roots. These evidences taken together strongly suggest a cross-talk between ethylene and auxin under hypoxic conditions, in which ethylene exerts a regulation on auxin levels/transport and on root growth and gravitropic responses through the control of the group VII ERF protein RAP2-12, a major regulator of low oxygen responses (Gibbs et al., 2011; Licausi et al., 2011; Eysholdt-Derzsó and Sauter, 2017). In parallel to the overall down-regulation of auxin, brassinosteroids and gibberellins biosynthesis, the biosynthesis of cytokinin and jasmonate appeared transcriptionally induced during waterlogging, suggesting that the latter two hormones may exert a further control over root growth (Wasternack and Hause, 2013). These changes, taken as a whole, suggest a highly co-ordinated reprogramming of the root hormonal profile which may fine-tune root growth and lateral root initiation in the presence of different oxygen availability. The significant enrichment of down-regulated DEGs in the GO category “regulation of meristem growth” may support an overall inhibition of root growth processes and lend further evidence to the hypothesis of a LOQS response taking place in K5BB roots exposed to low oxygen.

At 1 week after removal of the flooding stress (T6), most of these pathways resulted to be fully recovered at the transcriptional level, indicating a prompt readjustment of hormonal responses during the recovery phase. This was particularly evident for gibberellin biosynthetic genes that appeared down-regulated during flooding (T1 and T2) while those involved in metabolism were up-regulated. This effects appeared to be reversed as soon as the waterlogging ceased, indicating also a highly modulated control on gibberellin levels in relation to oxygen. The latter hormonal changes may be connected with the response of the upper, above ground, aerial plant. The inhibition of internode elongation soon after stress exposure (at the onset of budbreak) and the significant stimulation of internode elongation during later recovery time-points in plants that had experienced flooding in comparison to control ones are consistent with the timing of inactivation and reactivation of GA biosynthesis and action (Figure 9).

These findings are also in agreement with those reported by Stevens and Prior (1994) in potted Sultana vines during initial growth stages after budbreak with an initial shoot elongation rate of about 30% during the first 4 weeks after stress removal. Also the recovery of shoot elongation rates resulted in agreement with data available in the literature (Stevens and Prior, 1994; Stevens et al., 1999) reporting that the vines took more than 11 days to overcome flooding stress. In the present experiment, the inhibitory effect on shoot elongation lasted about 20 days after stress removal. Interestingly, budbreak in flooded plants appeared slightly but significantly slower than in non-flooded control ones. This early effect may also be related to the substantial hormonal changes leading to a likely abundance of ethylene in hypoxic roots immediately (within 1–2 days) followed by a likely accumulation of ACC, which could not be any more converted to the active hormone due to the lack of oxygen.

Additional experimental evidence will be needed to show how the main parental grapevine rootstock genotypes *V. riparia*, *V. rupestris* and *V. berlandieri* are affected by waterlogging in the short and long term to further clarify the role of the metabolic and transcriptomic reprogramming described in this work and to elucidate which processes are decisive in regulating flooding tolerance in grapevine. A comparison between a resistant and a sensitive genotype to waterlogging would thus enable to identify genotype-specific responses and to define key markers for tolerance to flooding that could be used to improve selection of waterlogging tolerant rootstocks. Furthermore, serious attention should be paid on that the effects of the hormonal rearrangement taking place in the roots, shown in this study, may differ significantly between different rootstocks genotypes. This may happen both during waterlogging or during the recovery phase, the latter one being an equally essential step defining plants adaptation and survival to flooding (Yeung et al., 2018), and may result in a range of phenological effects on the above ground plant, with varied viticultural implications.

## Data Availability

The datasets generated for this study can be found in NCBI BioProject database, PRJNA521303.

## Author Contributions

FM, BR, AB, and SQ developed the concept of the manuscript and wrote the manuscript. MB and AB performed the whole transcriptome and bioinformatic analyses. GE, FP, ST, and NC carried out qRT-PCR analyses and sample preparation. ES and PG carried out NMR metabolic profiling and FM collected and analyzed biometrical data. All authors discussed and commented on the manuscript.

## Conflict of Interest Statement

The authors declare that the research was conducted in the absence of any commercial or financial relationships that could be construed as a potential conflict of interest.

## Footnotes

1 https://www.lexogen.com/quantseq-data-analysis/

## Abbreviations

^1^H-NMR: proton nuclear magnetic resonance spectroscopy
ABA-GT: ABA glycosyltransferase
ACC: Aminocyclopropane-1-carboxylic acid
ACS: Aminocyclopropane-1-carboxylic acid synthase (ACC synthase)
ACX: Acetyl-coenzyme A oxidase
AGR: absolute growth rate
AHP: Histidine-containing phosphotransmitters
AO3: aldehyde oxidase
AP2/ERF: APETALA2/Ethylene-Responsive Factor
ARFs: Auxin Response Factors
ARR factors: transcription factors
ATP: adenosine triphosphate
C: control treatment
cDNA: complementary DNA
D_2_O: Deuterium oxide
DEGs: differentially expressed genes
DNase: deoxyribonuclease
DOY: day of the year
EBF 1/2: F-box proteins
ERF: ethylene responsive factor
ETR: ethylene response
F: flooding treatment
FDR: false discovery rate
FID: free induction decay
GABA: gamma amino butyric acid
GO: Gene Ontology
IPT: isopentenyltransferase
JAZ: jasmonate-zim-domain protein
K5BB: Kober 5BB
KEGG: Kyoto Encyclopedia of Genes and Genomes
LOES: Low Oxygen Escape Syndrome
LOQS: Low Oxygen Quiescence Syndrome
MeOH: methanol
MeOH-*d*_4_: deuterated methanol
MFP2: Peroxisomal fatty acid beta-oxidation multifunctional protein
n.d.: not detectable
NADH: Nicotinamide adenine dinucleotide hydride
NCED: 9-*cis*-epoxycarotenoid oxidases
NMR: Nuclear Magnetic Resonance
NOESY: nuclear overhauser effect spectroscopy
OPCL: Peptidoglycan-associated protein
OPLS-DA: supervised partial least squares discriminant analysis
PCA: principal component analysis
PDC: pyruvate decarboxylase
PIN: PIN-formed
PPi: inorganic pyrophosphate
RNA-seq: RNA sequencing
ROS: reactive oxygen species
RPKM: reads per kilobase million
RT-qPCR: real time quantitative polymerase chain reaction
SAUR: Small Auxin Up RNA
SDH: succinate dehydrogenase
T1: 1 day after stress application
T2: 2 days after stress application
T3: 8 days after stress application
T4: 16 days after stress application
T5: 21 days after stress application
T6: 7 days after stress removal
TCA cycle: tricarboxylic acid cycle
*VvACO2*: *VvACO1*, *Vitis vinifera* genes encoding for 1-aminocyclopropane-1-carboxylic acid oxidases
*VvADH1*: *Vitis vinifera* genes encoding for alcohol dehydrogenase
*VvSUS4*: *Vitis vinifera* genes encoding for sucrose synthase
*VvUBC28*: *Vitis vinifera* genes encoding for ubiquitin C.
