# Supplementary material for: Flooding Responses on Grapevine: A Physiological, Transcriptional, and Metabolic Perspective
Source: Front Plant Sci. 2019 Mar 26;10:339. doi: 10.3389/fpls.2019.00339 (PMC6443911; doi:10.3389/fpls.2019.00339)
Supplement: Supplementary file 1 [file Image_1.pdf]

Supplementary Material

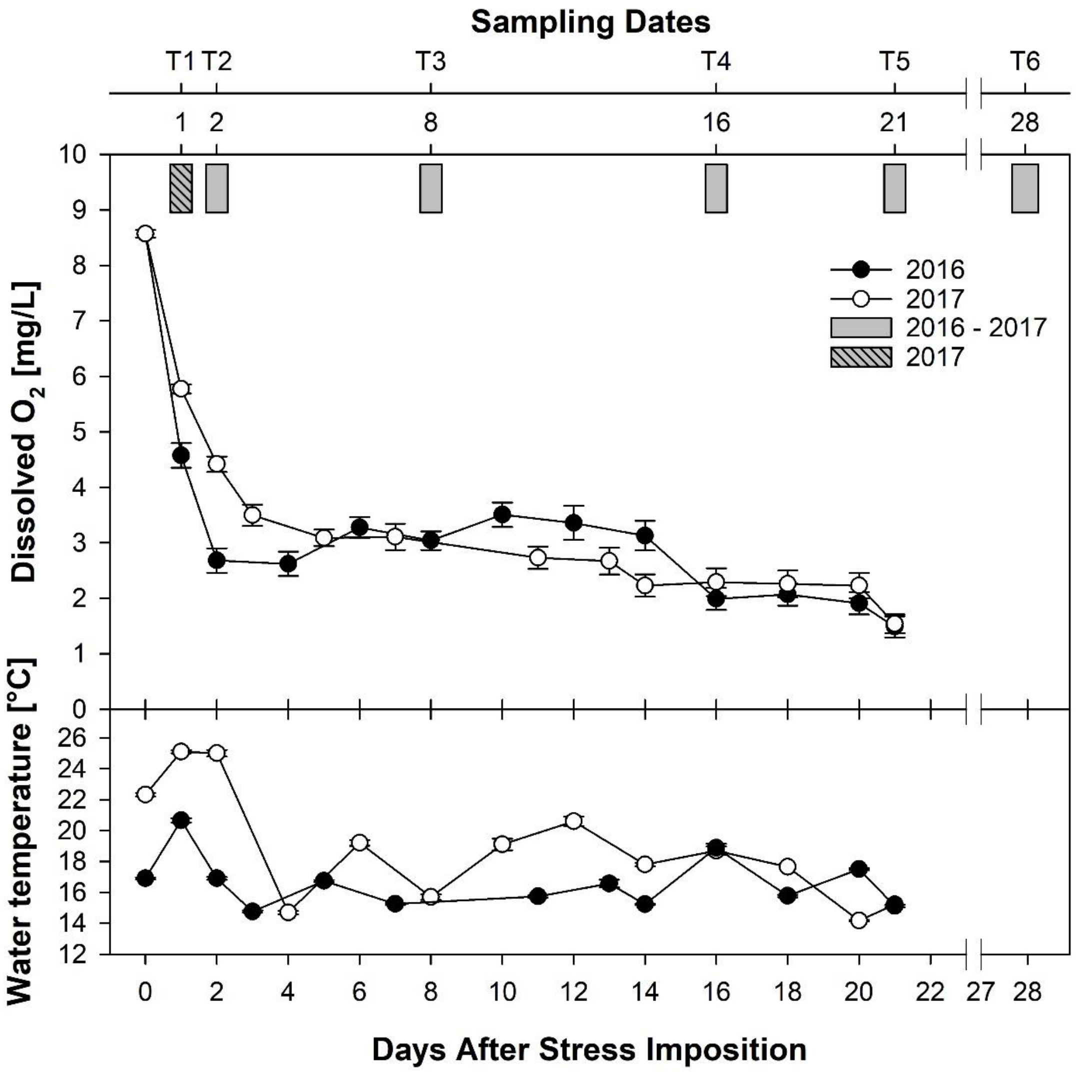

Supplementary Figure S1. Trend of dissolved O_2 concentration (mg/L) and water temperature (°C) during the 21 days of the pots experiment over the seasons 2016 (●) and 2017 (○). Grey bars indicate sampling points at 24h (T1, 2017 only), 2 (T2), 8 (T3), 16 (T4), 21 (T5) days after stress imposition. After the last sampling, the water was withdrawn from the pots and after 7 days (T6) an additional sampling of material was performed in both seasons.

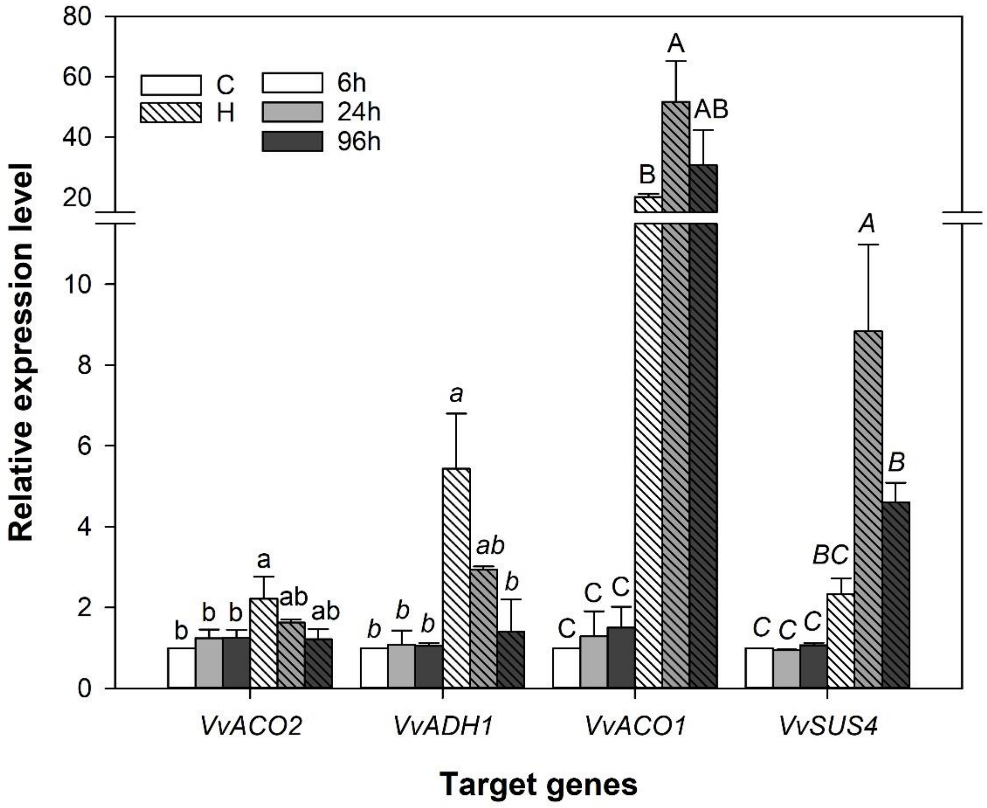

Supplementary Figure S2. Effect of control (C) and progressive hypoxic (H) treatments at 6h, 24h and 96h of stress imposition under hydroponic conditions on the relative expression of candidate hypoxia-responsive markers: *VvACO2*, *VvADH1*, *VvACO1* and *VvSUS4* in grapevine roots (K5BB). The values of the relative expression level is the mean ± SE of three independent biological replicates, and those values indicated with the same letters do not significantly differ according to LSD's test ($p < 0.05$).

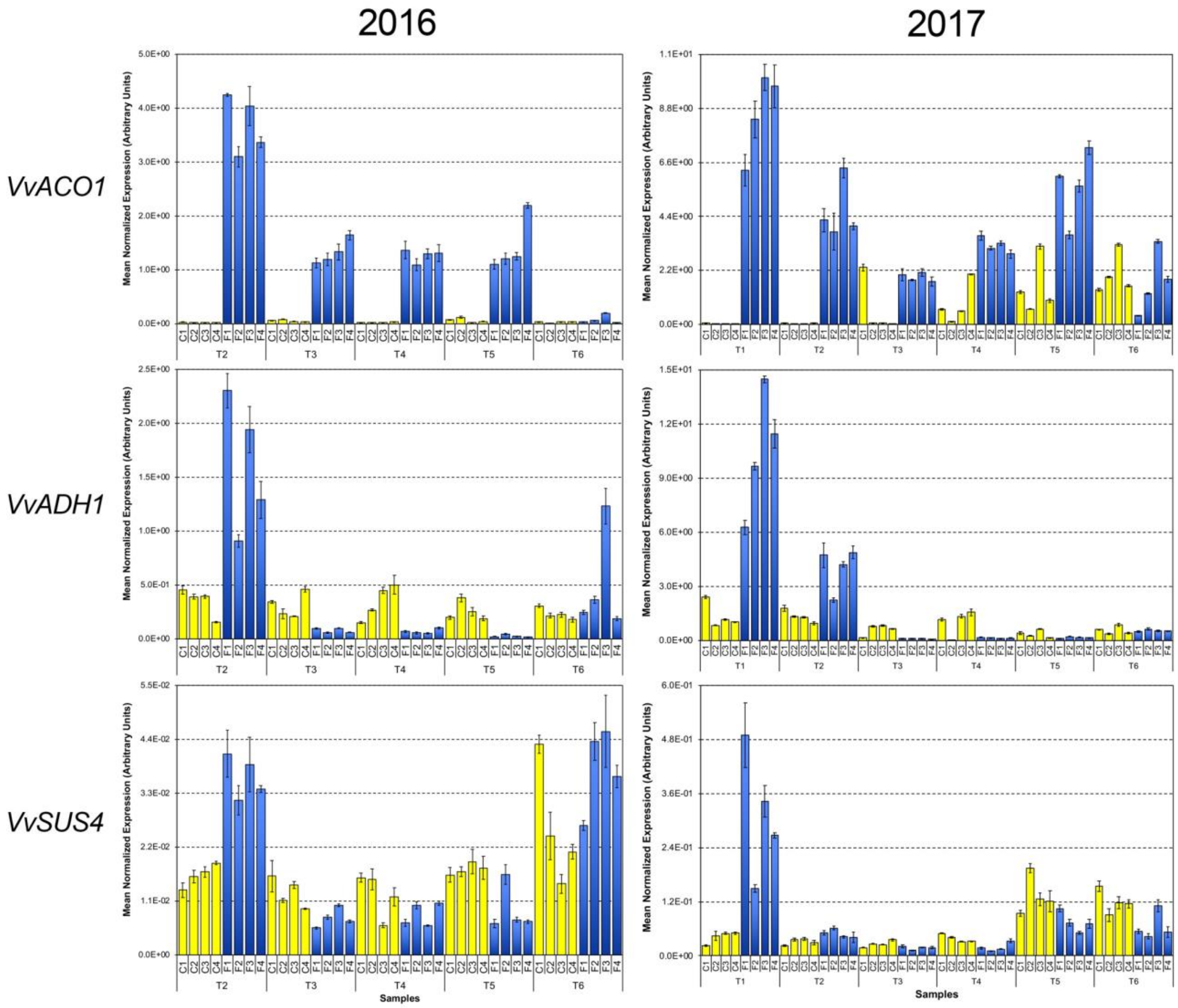

Supplementary Figure S3. RT-qPCR expression levels of the candidate hypoxia-responsive markers *VvACO1*, *VvADH*1 and *VvSUS4* in four independently biological replicates of roots from flooded (blue) versus control (yellow) plants evaluated in two consecutive years 2016 (left) and 2017 (right) at all the time points. The error bars rapresent the standard error. F=Flooded; C= Control.

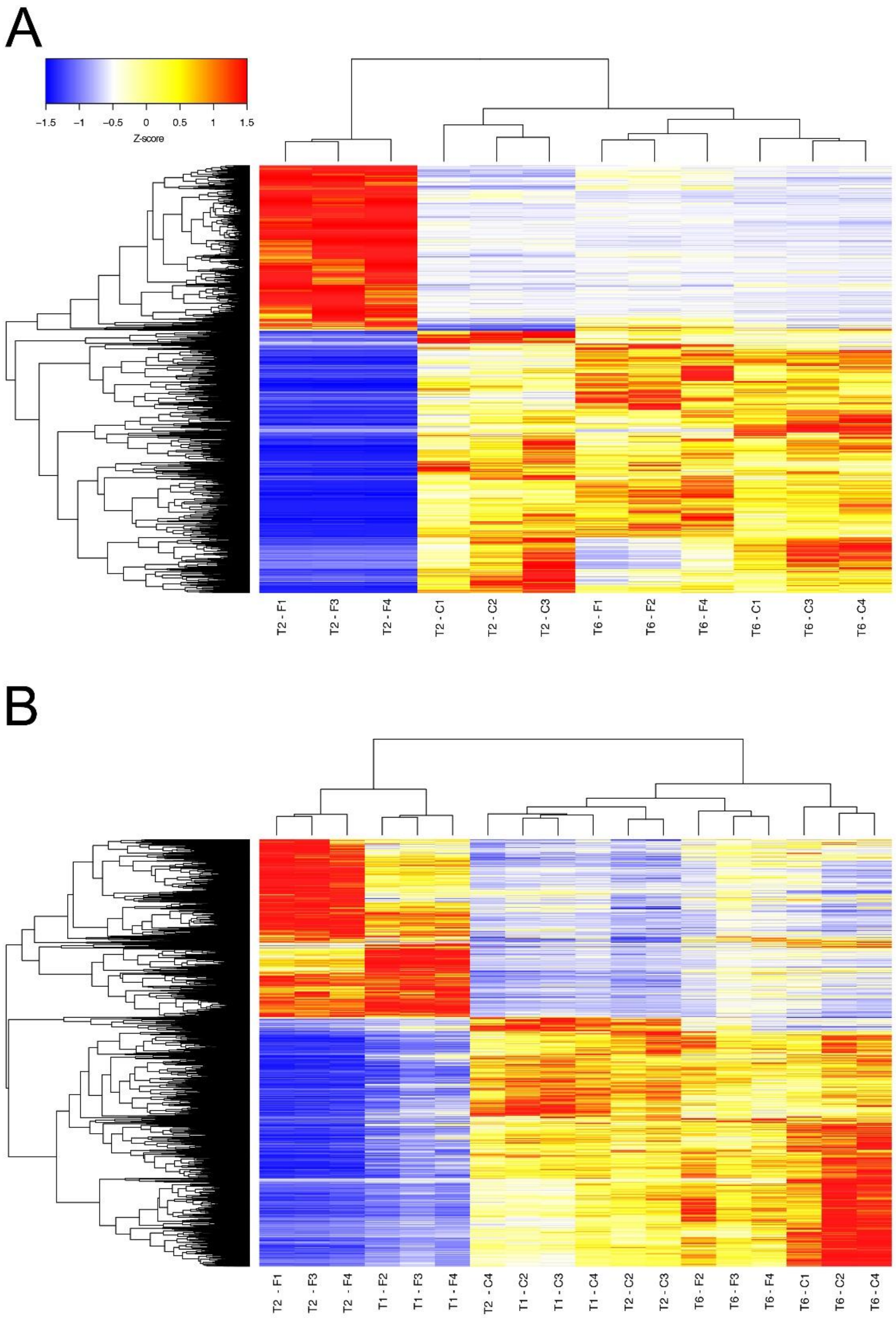

Supplementary Figure S4. Heatmaps showing separately the RNAseq transcriptional profiles of three biological replicates of roots of flooded (F) and control (C) plants, for the two years 2016 (A, top panel) and 2017 (B, bottom panel), respectively.

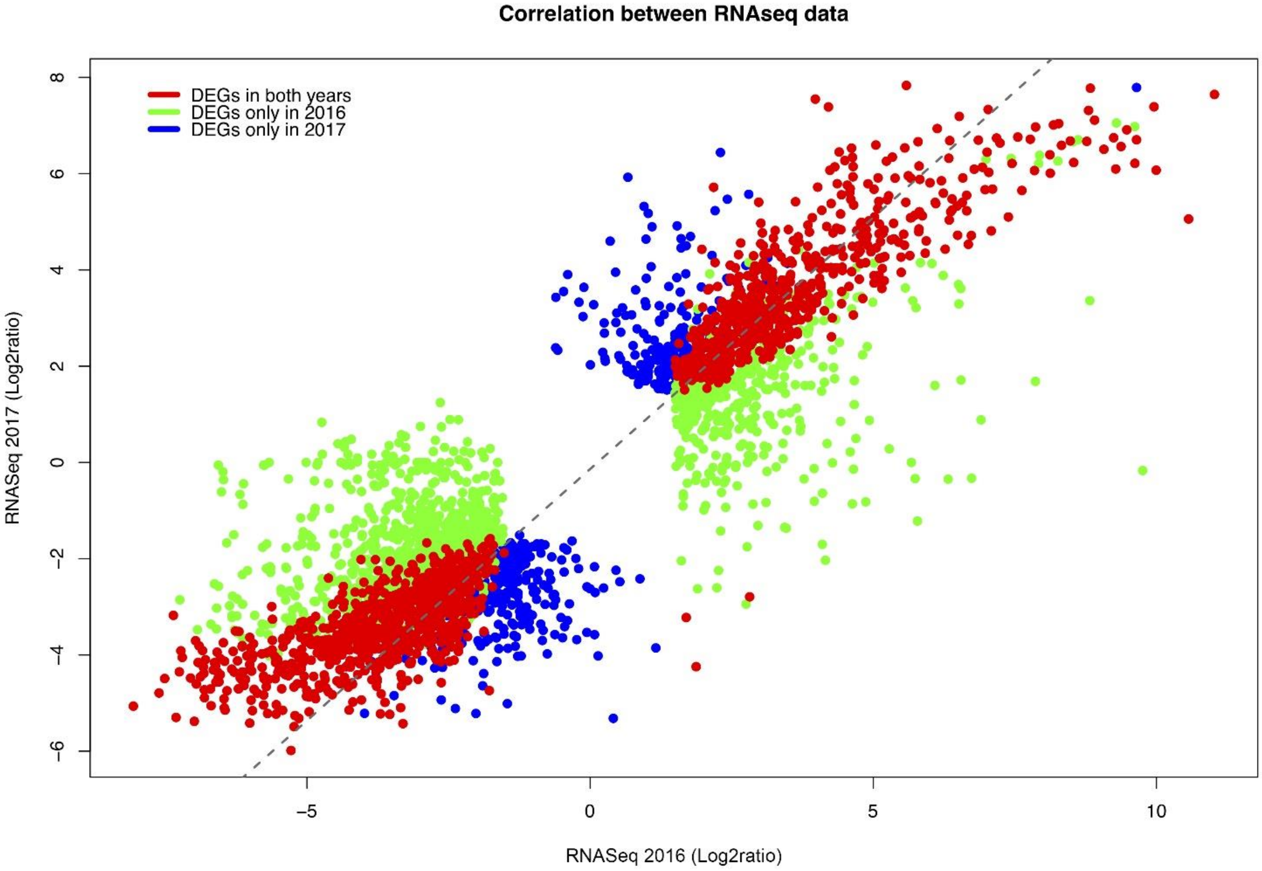

Supplementary Figure S5. Correlation among the differentially expressed genes (DEGs), calculated as log_2-ratio (Flooded versus Control) of expression data from RNAseq, in the two years 2016 and 2017. Red spots represent the DEGs conserved in both years.

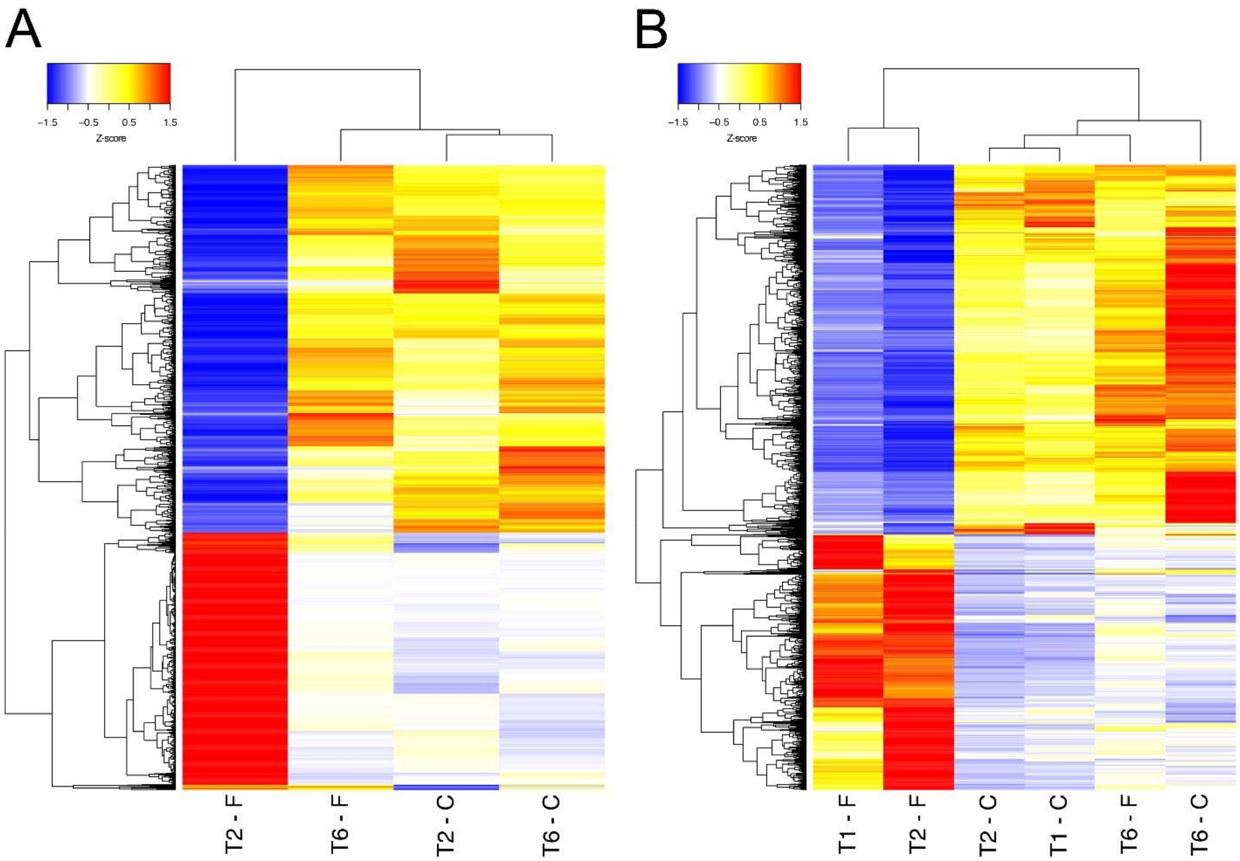

Supplementary Figure S6. Heatmaps showing the mean expression value avaraged betweem the three biological replicates of roots samples from flooded (F) and control (C) plants of the 2016 (A, left panel) and 2017 (B, right panel) years, respectively.

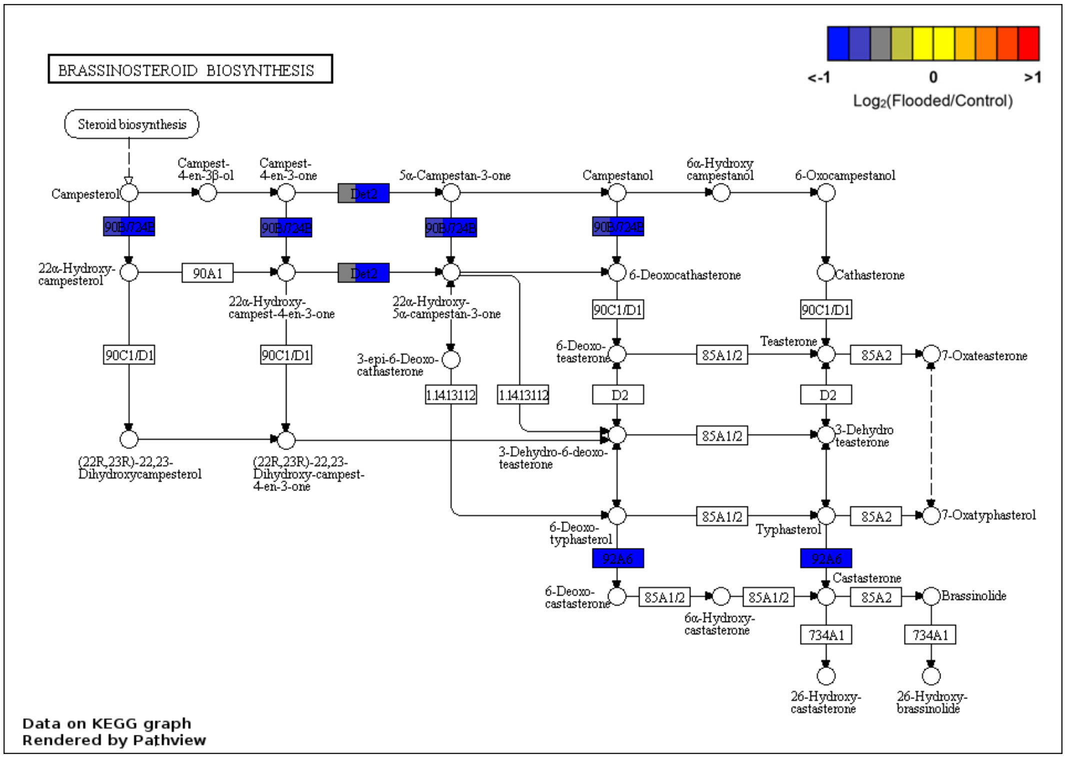

Supplementary Figure S7. KEGG map of Brassinosteroid biosynthesis (map00905) pathway, rendered by Pathview (Luo et al., 2017). Each box is divided in three sub-boxes and represents a specific enzymatic step with the relative expression of the corresponding genes in Flooded versus Control roots, calculated as log2-ratio at T1, T2 and T6 (from left to right).

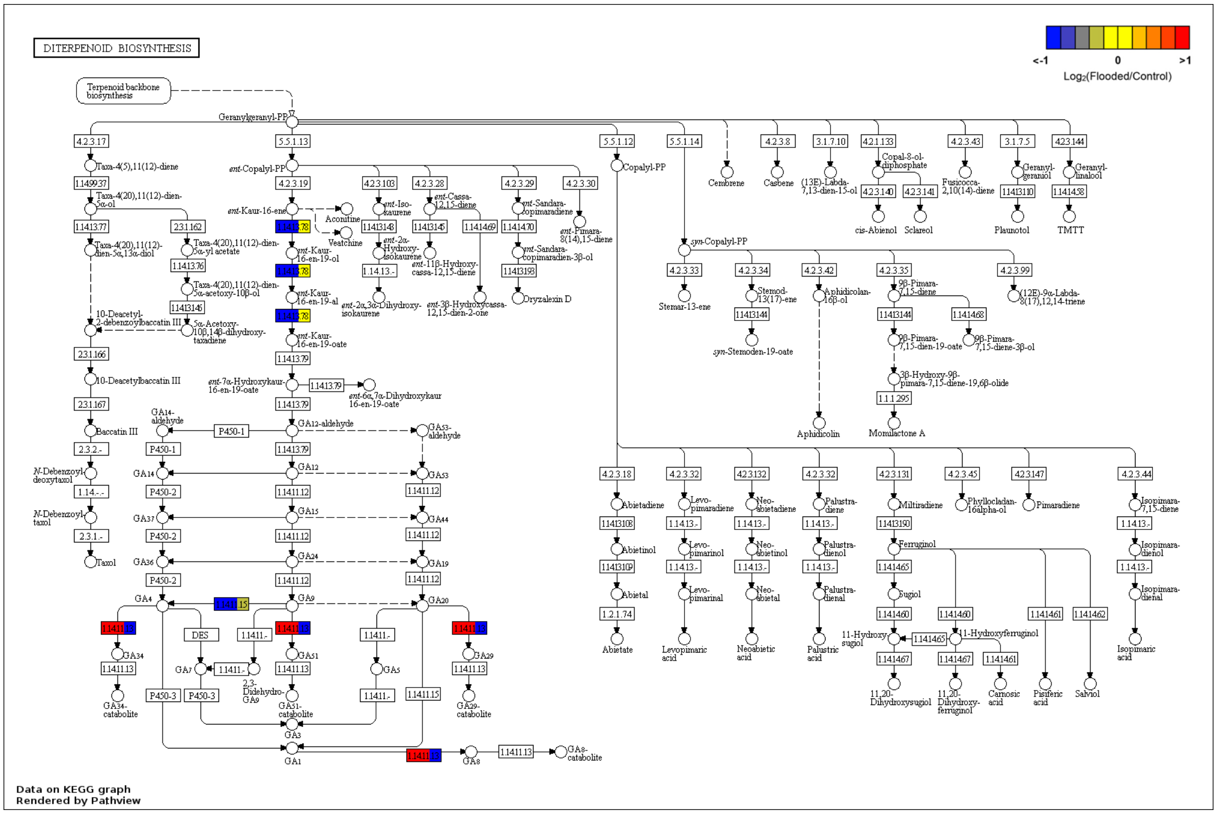

Supplementary Figure S8. KEGG map of Diterpenoid biosynthesis (map00904) pathway, rendered by Pathview (Luo et al., 2017). Each box is divided in three sub-boxes and represents a specific enzymatic step with the relative expression of the corresponding genes in Flooded versus Control roots, calculated as log2-ratio at T1, T2 and T6 (from left to right).

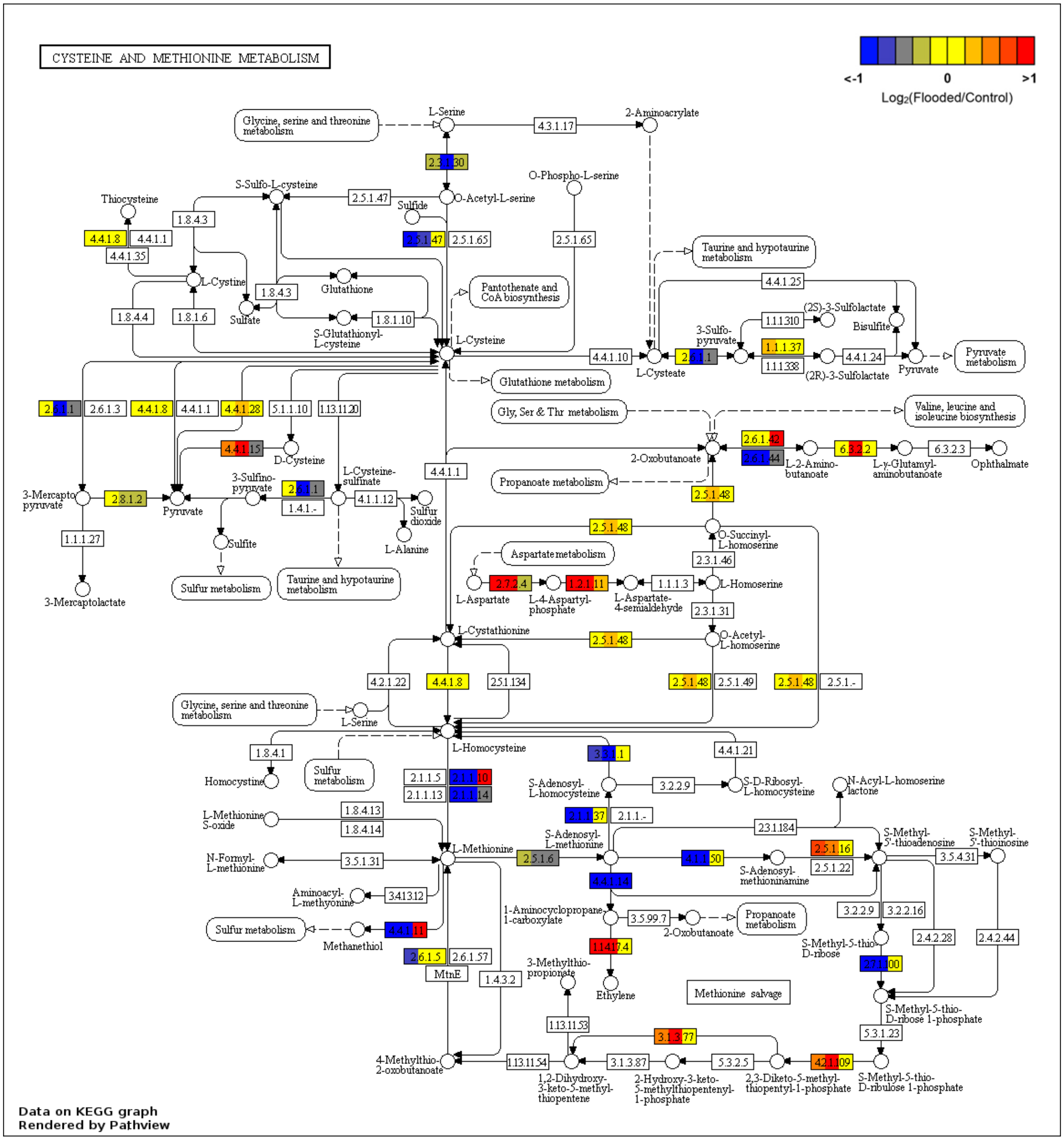

Supplementary Figure S9. KEGG map of Cysteine and Methionine metabolism (map00270) pathway, rendered by Pathview (Luo et al., 2017). Each box is divided in three sub-boxes and represents a specific enzymatic step with the relative expression of the corresponding genes in Flooded versus Control roots, calculated as log2-ratio at T1, T2 and T6 (from left to right).

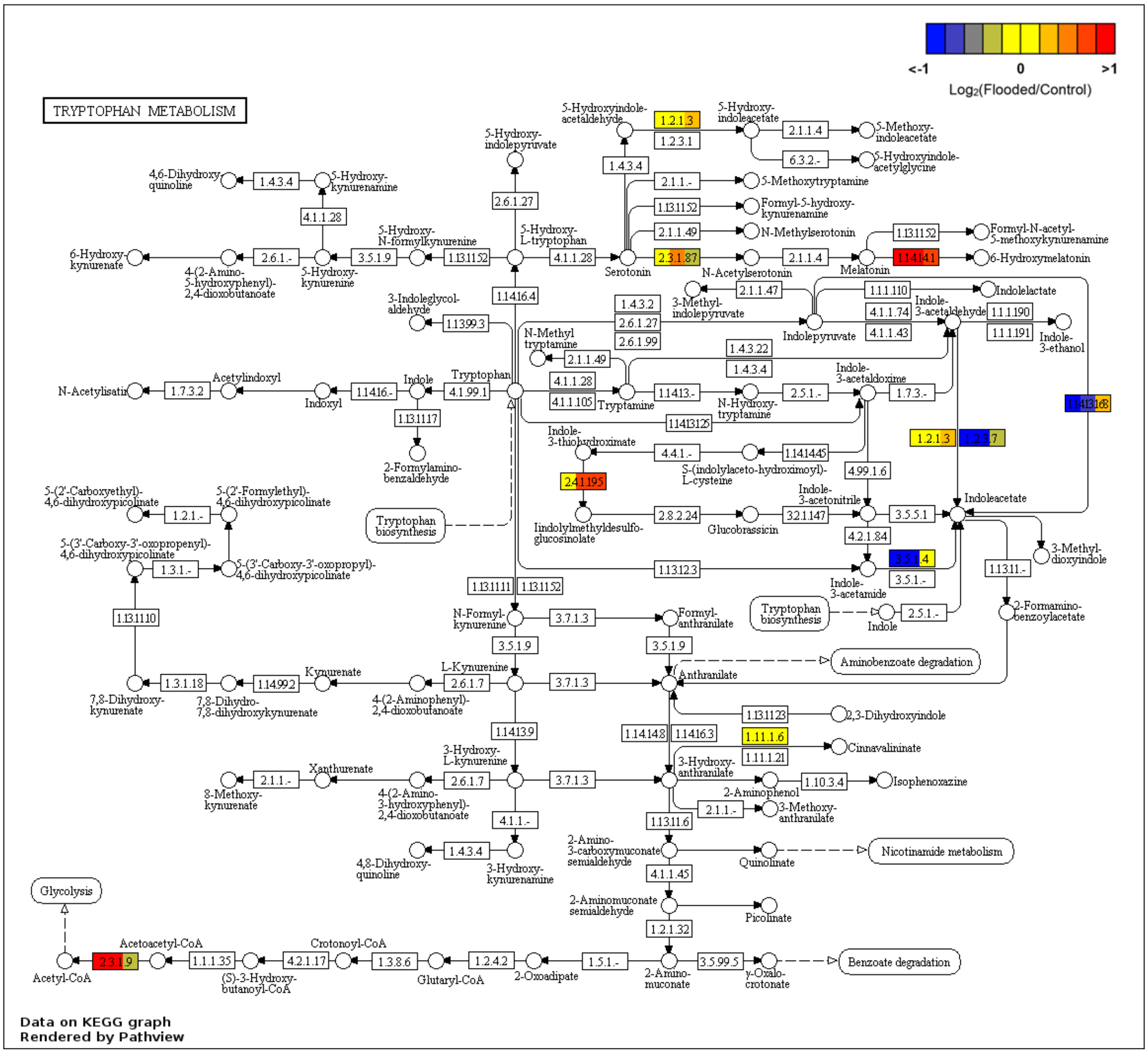

Supplementary Figure S10. KEGG map of Tryptophan metabolism (map00380) pathway, rendered by Pathview (Luo et al., 2017). Each box is divided in three sub-boxes and represents a specific enzymatic step with the relative expression of the corresponding genes in Flooded versus Control roots, calculated as log2-ratio at T1, T2 and T6 (from left to right).

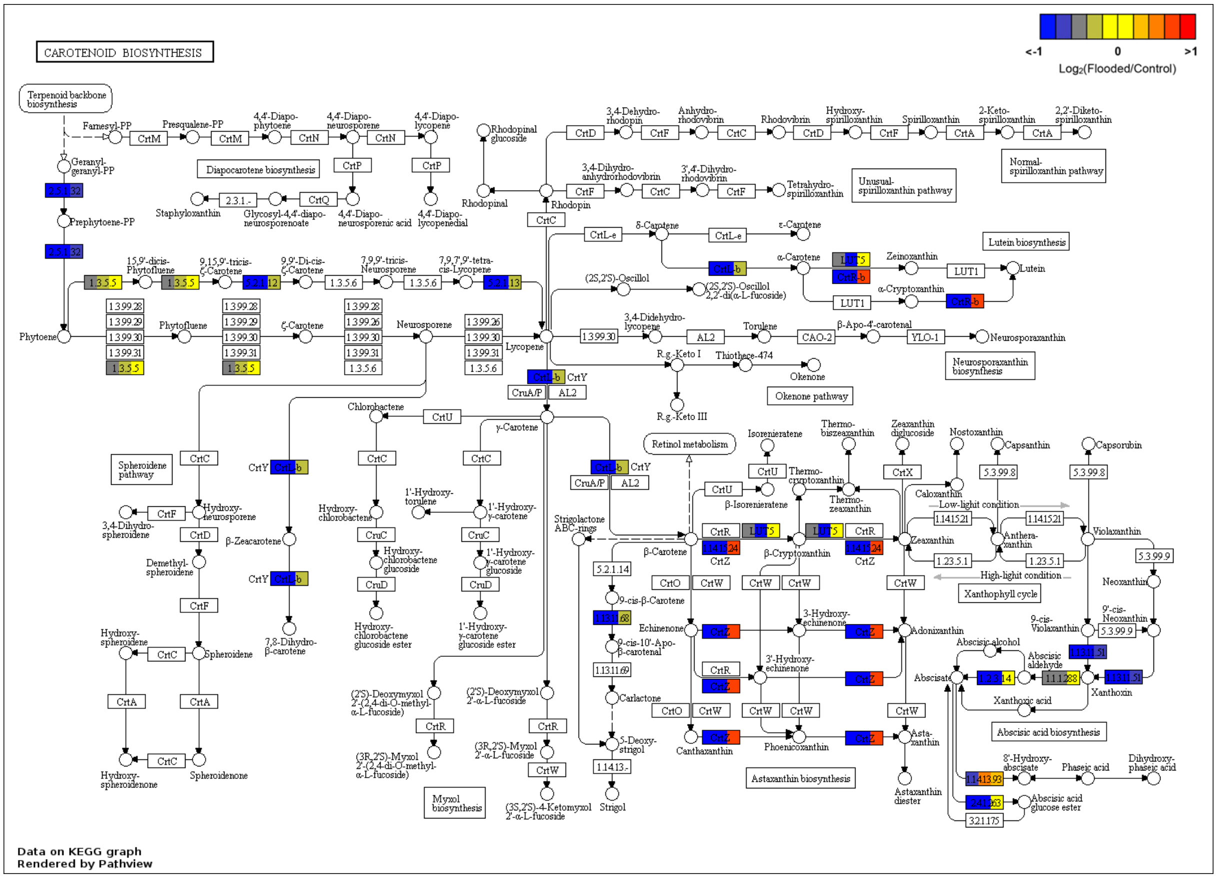

Supplementary Figure S11. KEGG map of Carotenoid biosynthesis (map00906) pathway, rendered by Pathview (Luo et al., 2017). Each box is divided in three sub-boxes and represents a specific enzymatic step with the relative expression of the corresponding genes in Flooded versus Control roots, calculated as log2-ratio at T1, T2 and T6 (from left to right).

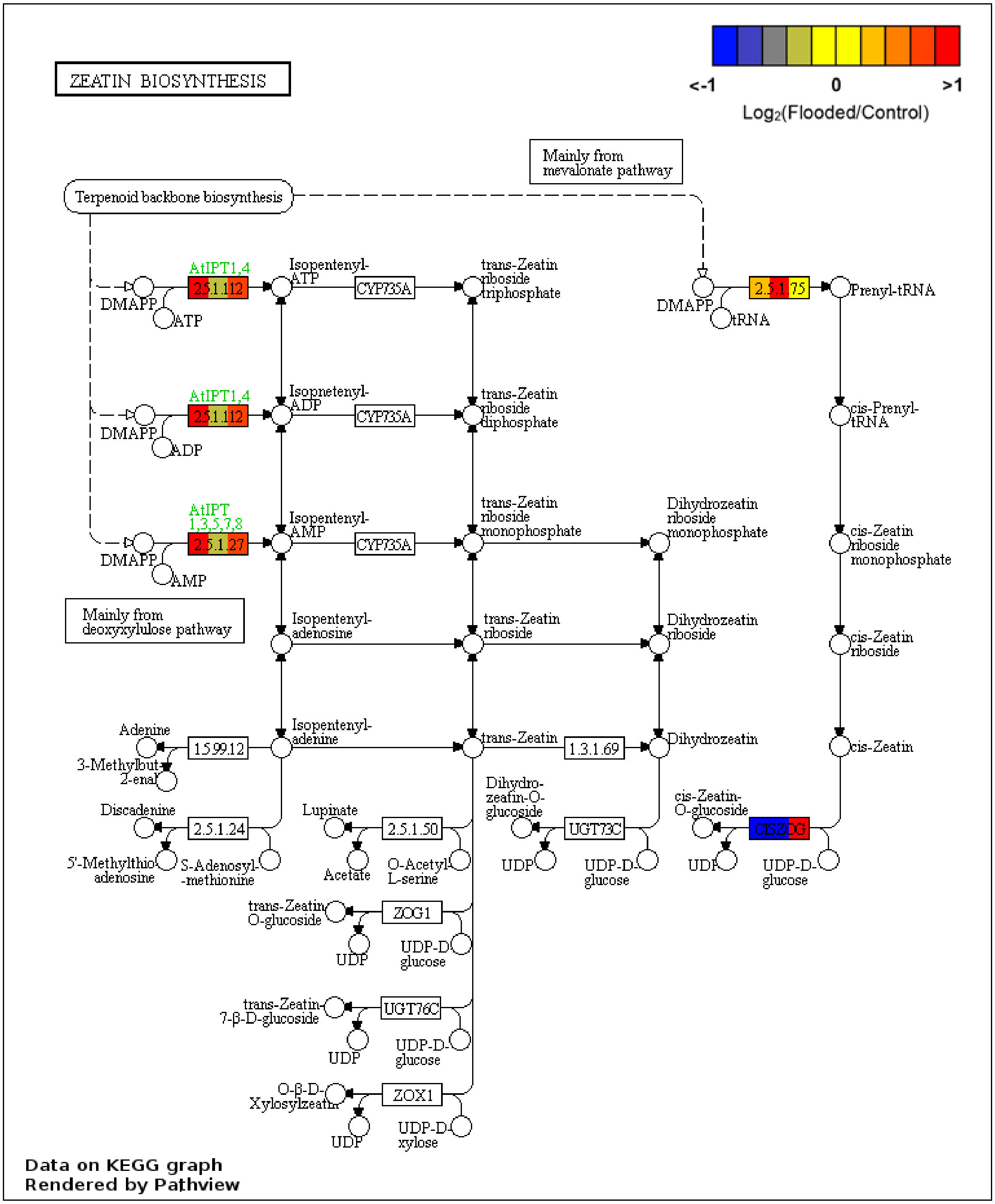

Supplementary Figure S12. KEGG map of Zeatin biosynthesis (map00908) pathway, rendered by Pathview (Luo et al., 2017). Each box is divided in three sub-boxes and represents a specific enzymatic step with the relative expression of the corresponding genes in Flooded versus Control roots, calculated as log2-ratio at T1, T2 and T6 (from left to right).

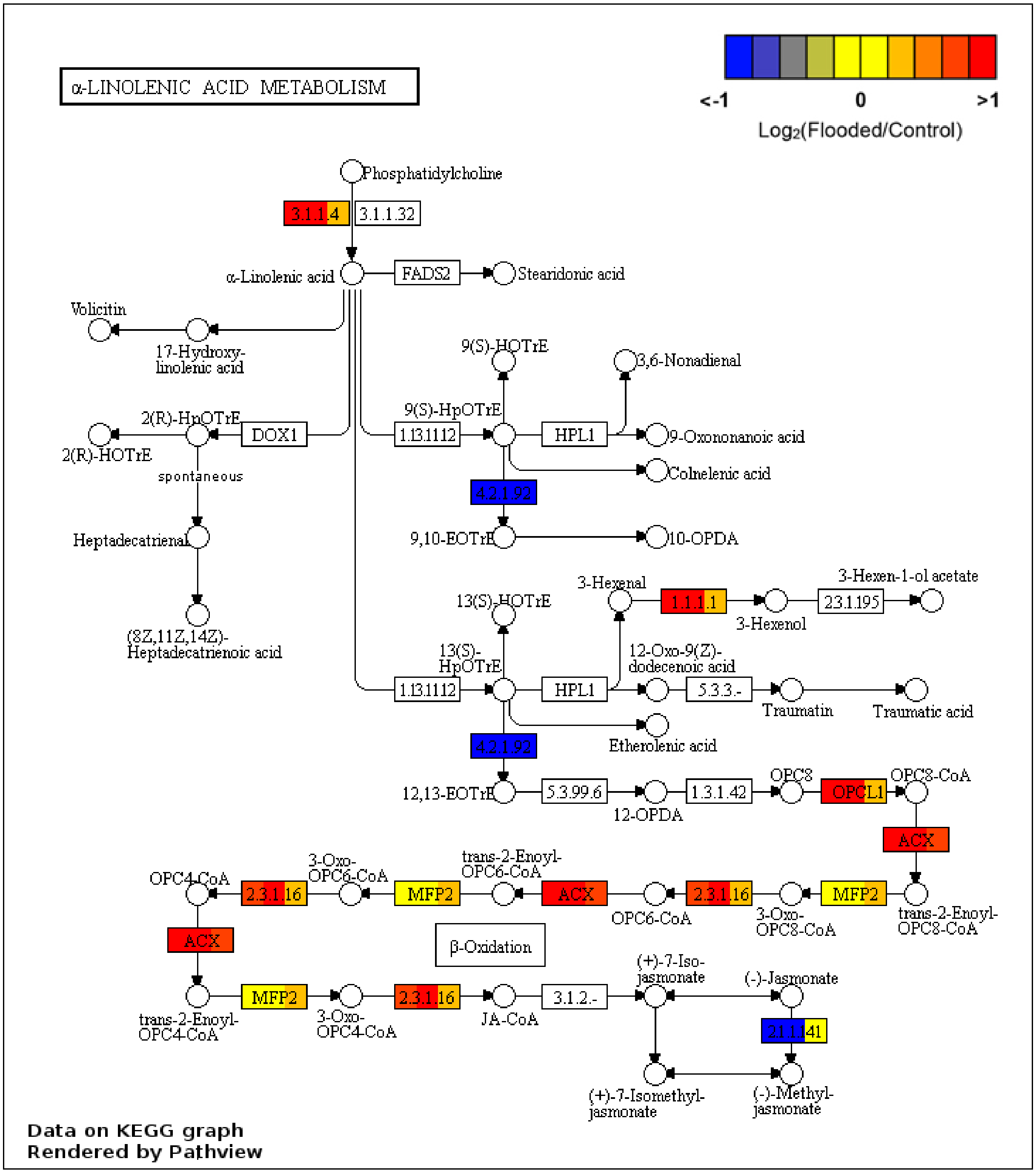

Supplementary Figure S13. KEGG map of α-Linoleinic acid metabolism (map00908) pathway, rendered by Pathview (Luo et al., 2017). Each box is divided in three sub-boxes and represents a specific enzymatic step with the relative expression of the corresponding genes in Flooded versus Control roots, calculated as log2-ratio at T1, T2 and T6 (from left to right).

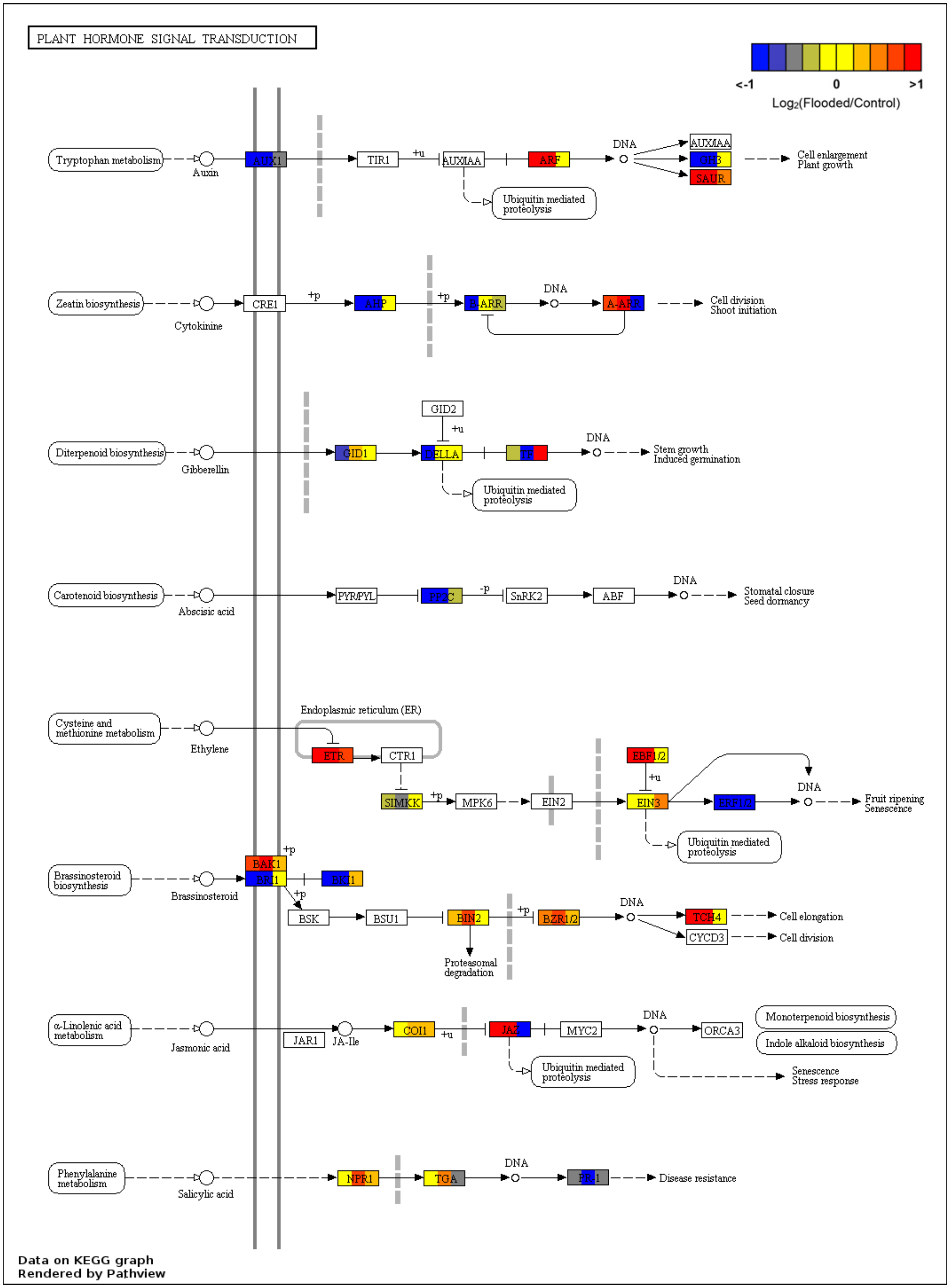

Supplementary Figure S14. KEGG map of Plant Hormone Signal Transduction (map04075) rendered by Pathview (Luo et al., 2017). Each box is divided in three sub-boxes and represents a specific step of the signal transduction pathway with the relative expression of

the corresponding genes in Flooded versus Control roots, calculated as log2-ratio at T1, T2 and T6 (from left to right).

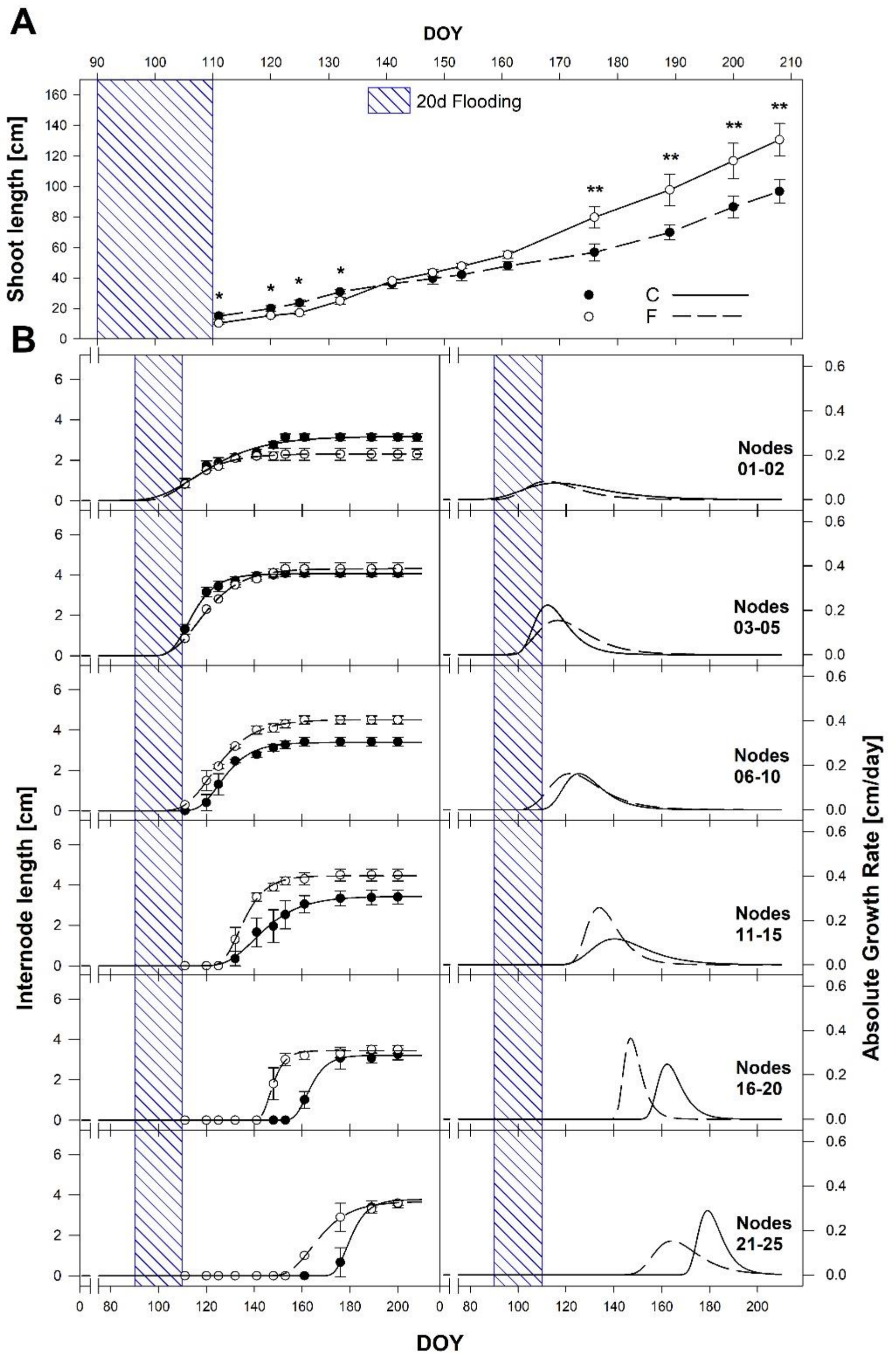

Supplementary Figure S15. A: Shoot length growth dynamics among control (C, black circles) and flooded (F, white circles) treatments throughout the 2016 season. **B**: Mean internode classes elongation dynamics: 1-2 (class I), 3-5 (class II), 6-10 (class III), 11-15 (class IV), 16-20 (class V) and 21-25 (class VI). Sigmoidal curves represent best fitting curves through nonlinear regression for C (solid line) and F (dotted line) treatments enabling absolute

growth rates dynamics (AGR) computation for each internode class. Asterisks corresponds to significant different among treatments (*, $p < 0.05$; **, $p < 0.01$).
