# Supplementary material for: Flooding Responses on Grapevine: A Physiological, Transcriptional, and Metabolic Perspective
Source: Front Plant Sci. 2019 Mar 26;10:339. doi: 10.3389/fpls.2019.00339 (PMC6443911; doi:10.3389/fpls.2019.00339)
Supplement: Supplementary file 2 [file Table_1.pdf]

Supplementary Material

Supplementary Table S1. Primers list used for RT-qPCR experiments

Gene Name	Primer Name	Primer Sequence	Gene ID (Locus Tag)	Reference
VvSUS4	VvSUS4_R<br>VvSUS4_F	GACAGTTATTCCACACAGCTCAGAAAACC<br>TGTCTCCAACCTTGACCGCCGTGA	VIT_211s0016g00470	In this article
VvACO2	VvACO_F<br>VvACO_R	GACTCCGAGCCCACACTGATGCCG<br>GGAGGCCGCTGACCGTGTCGTCTTG	VIT_212s0059g01380	Ziliotto *et al*. 2012
VvUBC28	VvUBC28_R<br>VvUBC28_F	AAGCCAGGCAGAGACAACTC<br>CTATATGCTCGCTGCTGACG	VIT_219s0015g01190	Castellarin *et al*. 2007
VvACO1	VvACO1_F<br>VvACO1_R	TTGGTTTGGAGAAGGGCTACATCCGAGAA<br>GCCTGGAACTTGATCATCTTGGAGCA	VIT_211s0016g02380	In this article
VvADH1	VvADH1_F<br>VvADH1_R	GTTGGGTGTTGCAGTAATTGTGGG<br>CGGAATATCAGAACGGGGCTTGTAGT	VIT_218s0001g15410	In this article

Supplementary Table S2. Output of the sequencing for samples taken in 2016

Sample name	# Total Reads	# Mapped Reads	% Mapped Reads
T2-C1	33,256,854	26,094,920	78.50%
T2-C2	34,183,433	28,815,874	84.30%
T2-C3	23,822,683	20,356,181	85.40%
T2-F1	32,914,274	27,417,150	83.30%
T2-F3	33,803,128	27,061,967	80.10%
T2-F4	34,844,676	27,364,851	78.50%
T6-C1	34,888,771	28,240,980	80.90%
T6-C3	34,317,974	27,654,158	80.60%
T6-C4	33,824,614	26,032,747	77.00%
T6-F1	35,793,713	27,864,385	77.80%
T6-F2	30,162,961	26,542,949	88.00%
T6-F4	32,740,082	28,807,872	88.00%

Supplementary Table S3. Output of sequencing for samples taken in 2017

Sample name	# Total Reads	# Mapped Reads	% Mapped Reads
T1-C2	18,025,490	12,231,172	67.90%
T1-C3	17,685,164	12,433,356	70.30%
T1-C4	21,497,367	14,851,717	69.10%
T1-F2	16,900,207	11,880,394	70.30%
T1-F3	26,704,229	17,091,423	64.00%
T1-F4	14,888,011	9,820,927	66.00%
T2-C2	21,421,112	14,737,392	68.80%
T2-C3	17,047,963	12,460,595	73.10%
T2-C4	19,869,262	13,839,799	69.70%
T2_F1	23,666,315	15,487,009	65.40%
T2-F3	18,196,938	12,596,366	69.20%
T2-F4	18,566,705	12,433,038	67.00%
T6-C1	22,262,353	14,524,367	65.20%
T6-C2	19,764,266	11,887,037	60.10%
T6-C4	17,656,854	11,326,103	64.10%
T6-F2	22,523,198	15,252,157	67.70%
T6-F3	21,785,960	13,531,500	62.10%
T6-F4	18,666,336	13,255,603	71.00%

Supplementary Table S4. Fit parameters and goodness of fit for the internode growth models

Year	Treatment	Internode class	Gompertz three-parameter model						Goodness of fit	
			a		*b*		x_0		r^2	SEE
2016	C	01-02	3.18	± 0.08	15.73	± 1.95	115.25	± 1.47	0.98	0.15
	F		2.31	± 0.02	10.27	± 0.47	111.44	± 0.49	0.99	0.04
	C	03-05	4.05	± 0.03	6.68	± 0.57	111.78	± 0.82	1.00	0.09
	F		4.31	± 0.04	10.25	± 0.7	115.94	± 0.59	1.00	0.09
	C	06-10	3.39	± 0.05	7.65	± 0.72	124.87	± 0.5	0.99	0.11
	F		4.51	± 0.03	10.18	± 0.42	121.23	± 0.27	1.00	0.06
	C	11-15	3.44	± 0.06	10.87	± 0.95	139.71	± 0.7	0.99	0.12
	F		4.45	± 0.04	6.36	± 0.35	133.35	± 0.28	1.00	0.08
	C	16-20	3.18	± 0.03	4.91	± 0.44	163.11	± 0.63	1.00	0.07
	F		3.44	± 0.05	3.46	± 0.54	146.48	± 0.41	1.00	0.10
	C	21-25	3.78	± 0.06	4.79	± 0.45	178.62	± 0.36	1.00	0.07
	F		3.68	± 0.07	8.88	± 0.78	163.69	± 0.58	1.00	0.09
2017	C	01-02	2.97	± 0.02	1.49	± 0.15	104.35	± 0.16	1.00	0.05
	F		2.30	± 0.01	2.05	± 0.13	101.63	± 0.12	1.00	0.02
	C	03-05	5.75	± 0.03	1.97	± 0.12	105.24	± 0.19	1.00	0.10
	F		3.83	± 0.02	2.50	± 0.34	104.15	± 0.65	1.00	0.05
	C	06-10	3.53	± 0.03	8.26	± 0.53	112.10	± 0.4	1.00	0.08
	F		3.38	± 0.04	8.07	± 0.75	115.62	± 0.54	0.99	0.11
	C	11-15	3.36	± 0.04	5.26	± 0.62	136.21	± 0.38	1.00	0.09
	F		4.02	± 0.04	2.91	± 0.78	135.12	± 0.28	1.00	0.08
	C	16-20	3.46	± 0.0006	5.67	± 0.01	148.99	± 0.01	1.00	0.00
	F		3.98	± 0.01	5.87	± 0.19	144.57	± 0.12	1.00	0.03
	C	21-25	3.49	± 0.02	5.51	± 0.26	160.26	± 0.19	1.00	0.04
	F		4.12	± 0.003	5.75	± 0.06	156.02	± 0.03	1.00	0.01

Final shoot length, internode elongation duration and maximum internode elongation rate were estimated for five classes of internodes: from basal (1-2, 3-5), to median (6-10, 11-15, 16-20) to apical (21-25) by non-linear least square regression of the change in internode length on a day of the year (DOY) basis, using a three-parameter Gompertz sigmoidal model as follow:

$$Internode\ length = a \cdot e^{-e^{-\left(\frac{x-x_0}{b}\right)}}$$

where: a = final internode length; x_0 = the x-value at the inflexion point corresponding to the midway point in the duration of internode expansion; b = is a growth-rate coefficient; r^2= coefficient of determination; SEE = standard error of estimate.
